# Membrane Associated RNA‐Containing Vesicles Regulate Cortical Astrocytic Microdomain Calcium Transients in Awake Ischemic Stroke Mice

**DOI:** 10.1002/advs.202404391

**Published:** 2024-10-23

**Authors:** Zhongqiu Zhou, Ying Bai, Xiaochun Gu, Hui Ren, Wen Xi, Yu Wang, Liang Bian, Xue Liu, Ling Shen, Xianyuan Xiang, Wenhui Huang, Zhuojuan Luo, Bing Han, Honghong Yao

**Affiliations:** ^1^ Department of Pharmacology Jiangsu Provincial Key Laboratory of Critical Care Medicine School of Medicine Southeast University Nanjing 210009 China; ^2^ Jiangsu Key Laboratory of Molecular and Functional Imaging Department of Radiology Zhongda Hospital Medical School of Southeast University Nanjing 210009 China; ^3^ Shenzhen Institute of Advanced Technology Chinese Academy of Sciences Shenzhen 518055 China; ^4^ Department of Molecular Physiology Center for Integrative Physiology and Molecular Medicine University of Saarland 66421 Homburg Germany; ^5^ The Key Laboratory of Developmental Genes and Human Disease School of Life Science and Technology Southeast University Nanjing 210096 China; ^6^ Co‐innovation Center of Neuroregeneration Nantong University Nantong 226001 China; ^7^ Institute of Life Sciences Key Laboratory of Developmental Genes and Human Disease Southeast University Nanjing 210096 China

**Keywords:** astrocytic microdomain calcium transients, circSCMH1, ischemic stroke, membrane associated RNA‐containing vesicles, mitochondria

## Abstract

Astrocytic processes minutely regulate neuronal activity via tripartite synaptic structures. The precision‐tuning of the function of astrocytic processes is garnering increasing attention because of its significance in promoting brain repair following ischemic stroke. Microdomain calcium (Ca^2+^) transients in astrocytic processes are pivotal for the functional regulation of these processes. However, the understanding of the alterations and regulatory mechanism of microdomain Ca^2+^ transients during stroke remains limited. In the present study, a fast high‐resolution, miniaturized two‐photon microscopy is used to show that the levels of astrocytic microdomain Ca^2+^ transients are significantly reduced in the peri‐infarct area of awake ischemic stroke mice. This finding correlated with the behavioral deficits shown by these mice under freely‐moving conditions. Mitochondrial Ca^2+^ activity is an important factor driving the microdomain Ca^2+^ transients. DEAD Box 1 (DDX1) bound to circSCMH1 (a circular RNA involved in vascular post‐stroke repair) facilitates the formation of membrane‐associated RNA‐containing vesicles (MARVs) and enhances the activity of astrocytic microdomain Ca^2+^ transients, thereby promoting behavioral recovery. These results show that targeting astrocytic microdomain Ca^2+^ transients is a potential therapeutic approach in stroke intervention.

## Graphical abstract

Astrocytic microdomain Ca^2+^ transients diminish after ischemic stroke, which occur in peri‐synaptic astrocyte processes and end‐feet. Impaired motor behavior coincides with reduced microdomain Ca^2+^ transients in freely moving mice after stroke. The membrane associated RNA‐containing vesicles (MARVs) facilitate distribution of Ca^2+^ to mitochondria and increase astrocytic microdomain Ca^2+^ transients after stroke.

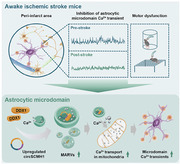

## 1 Introduction

Ischemic stroke is a prevalent neurologic condition and a major contributor to long‐term disability.^[^ ^1^ ^]^ Nevertheless, effective drugs that can promote brain repair after ischemic injury are currently lacking in clinical practice. Because therapies targeting exclusively neurons show suboptimal clinical benefits, studies examining astrocytes have been garnering increasing attention. The highly dynamic and plastic astrocytic processes constitute 75–85% of astrocytic volume and form a dense meshwork, and numerous peripheral synapses and capillaries are embedded.^[^ ^2^, ^3^ ^]^ Astrocytic processes act as signal “sensors” for neurovascular function and as “receptors” for the detection of changes in peripheral neuronal activity, oxygen and carbon dioxide levels, blood flow alterations, and other signal variations.^[^ ^4^, ^5^, ^6^ ^]^ A comprehensive understanding of the activity characteristics of astrocytes in the brain active milieu is essential for elucidating stroke progression.

Astrocytes expeditiously transmit signals via intracellular calcium (Ca^2+^) transport.^[^ ^3^, ^7^ ^]^ Recent advances in imaging technology and analysis algorithms have enabled detailed exploration of astrocytic Ca^2+^ transients in both spatial and temporal dimensions.^[^ ^8^, ^9^, ^10^, ^11^ ^]^ Local Ca^2+^ transients, known as microdomain Ca^2+^ transients, occur in astrocytic processes and end‐feet. These microdomain Ca^2+^ transients show characteristics that are distinct from soma Ca^2+^ transients, which manifest as smaller amplitude and higher frequency Ca^2+^ transients.^[^ ^12^, ^13^, ^14^ ^]^ Astrocytic microdomain Ca^2+^ transients are closely linked to neuronal activity and blood‐flow changes over specific time scales,^[^ ^15^, ^16^, ^17^ ^]^ highlighting their critical role in brain function. However, the characteristics and precise mechanisms of changes in astrocytic microdomain Ca^2+^ transients, especially in awake post‐stroke mice, remain unclear.

A substantial body of evidence indicates that the dynamics of mitochondria in astrocytic processes are closely related to microdomain Ca^2+^ transients.^[^ ^18^, ^19^ ^]^ Ca^2+^, released via instantaneous opening of the mitochondrial permeability transition pore (mPTP), is one of the primary sources of Ca^2+^ transients in the microdomain.^[^ ^20^ ^]^ Recent studies have shown that the precise spatial distribution of Ca^2+^ in the inner mitochondrial regions can be regulated by membrane associated RNA‐containing vesicles (MARVs), which possess an RNA core and are enriched in the DEAD Box 1 (DDX1) protein.^[^ ^21^ ^]^ However, whether MARVs are involved in the regulation of astrocytic microdomain Ca^2+^ transients, and what type of RNA participates in this process, require further investigation.

In the present study, we used a fast high‐resolution, miniaturized two‐photon microscope (mTPM) and in vivo Ca^2+^ labeling in astrocytes to determine that the astrocytic microdomain Ca^2+^ transients were diminished in the peri‐infarct area of freely‐behaving ischemic stroke mice. Real‐time monitoring of astrocytic microdomain Ca^2+^ transients in ischemic stroke mice during the performance of behavioral tasks indicated that the reduction in astrocytic microdomain Ca^2+^ transients was synchronous with behavioral deficits. Additionally, our results indicate that DDX1 coupled with circSCMH1, which enhanced the transport of Ca^2+^ from MARVs to the mitochondria and increased the microdomain Ca^2+^ transients. However, astrocyte‐specific downregulation of DDX1 expression inhibited the formation of MARVs, diminished the microdomain Ca^2+^ transients, and reduced the efficacy of extracellular vesicle (EV) delivery of circSCMH1 in enhancing post‐stroke recovery. The findings obtained in the present study provide basis for novel approaches in the promotion of recovery after stroke.

## 2 Results

### 2.1 Alterations in Astrocytic Microdomains are Dynamically Manifested After Stroke

**Figure 1:**
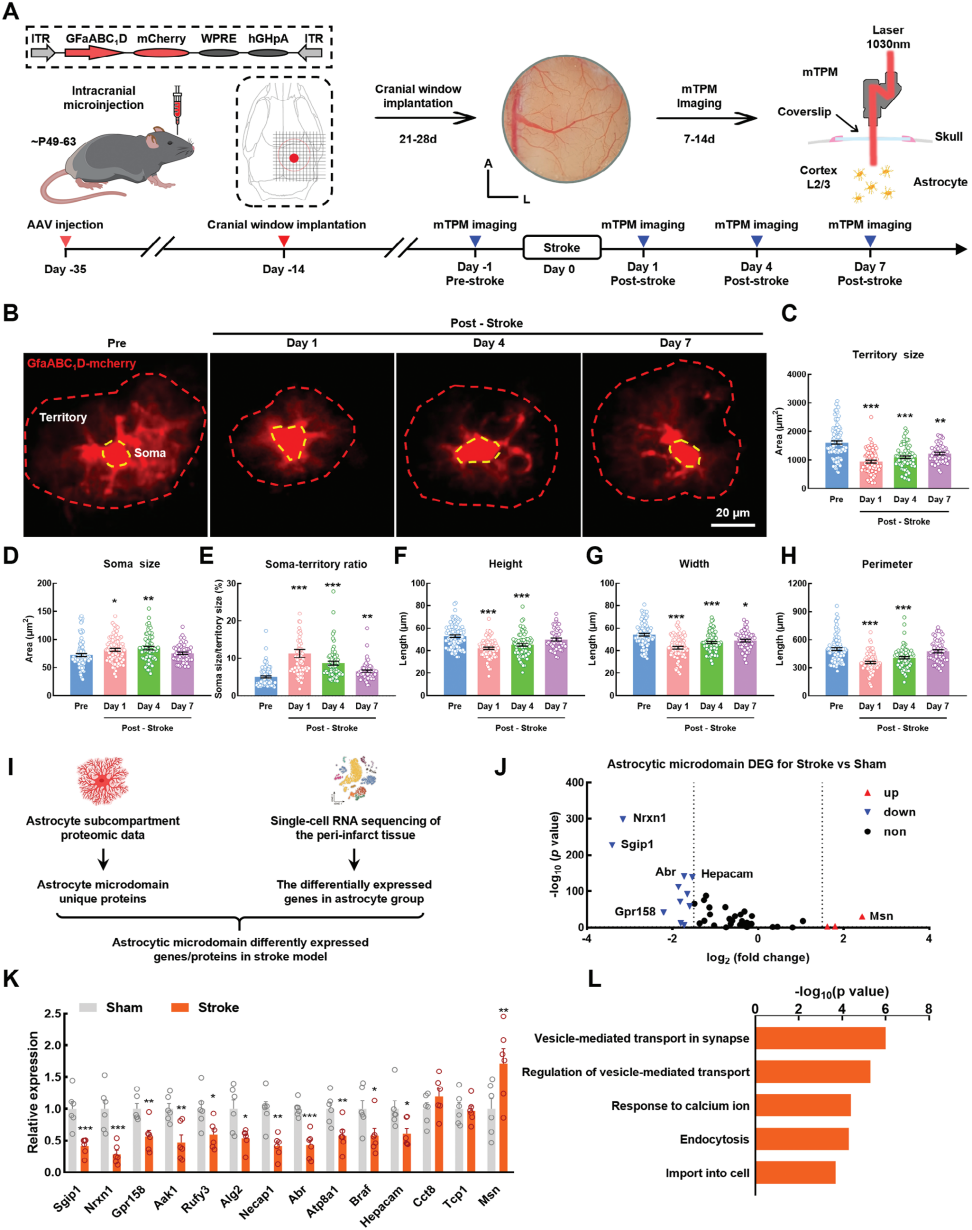
Alterations in astrocytic microdomains are dynamically manifested after stroke. A) Schematic of the approach for imaging of astrocytic morphology in vivo. Left: Cerebral cortex AAV injection for expressing mCherry in astrocyte. Middle: A cranial window was prepared for head‐fixed imaging with a hole (diameter = 5.0 mm). Right: Astrocyte morphological imaging with mTPM. mTPM: mini two‐photon microscope. Down: Illustration of the experimental procedure. B) Representative images of single astrocytes from same field of view in same mice peri‐infarct area at ‐1, 1, 4, 7d after stroke. C–H) Summary plots of the astrocyte morphological parameters: territory size (C), soma size (D), territory‐to‐soma ratio (E), height (F), width (G), perimeter (H). Pre = 90 astrocytes, Day 1 = 76 astrocytes, Day 4 = 67 astrocytes, Day 7 = 50 astrocytes. Data are shown as mean ± SEM. ^*^ *P* < 0.05, ^**^ *P* < 0.01, ^***^ *P* < 0.001 versus the Pre group using one‐way ANOVA followed by the Holm–Sidak post hoc multiple comparisons test. I) Schematic diagram of astrocytic microdomain different expression genes in stroke model. J) Volcano plot showing significantly differentially expressed genes (*P* < 0.01, |log_2_(fold change)| >1.5). K) qRT‐PCR validation of 14 genes differentially expressed in peri‐infarct area. *n* = 6 mice per group. Data are shown as mean ± SEM. ^*^ *P *< 0.05, ^**^ *P *< 0.01, ^***^ *P *< 0.001 versus the sham group using the Mann‐Whitney *U* test. L) Bar graph showing the top five significant GO terms in protein‐protein interaction enrichment analysis. Pre: Pre‐stroke; DEGs: differentially expressed genes.

Morphologic alterations in glial cells within the peri‐infarct area are usually manifested after stroke as soma enlargements and swelling of major branches.^[^ ^22^, ^23^ ^]^ However, vascular injury typically occurs with retraction or separation of astrocytic end‐feet from the blood vessels during stroke, which leads to a reduction in astrocyte volume.^[^ ^24^, ^25^ ^]^ Thus, the dynamic alterations in astrocyte morphology following stroke, especially changes in the fine processes, need to be further elucidated. In this study, we used the *GfaABC1D* promoter via an adeno‐associated virus (AAV) to express the mCherry protein in astrocytes. The morphologic changes in astrocytes were dynamically tracked by two‐photon microscopy using a chronically implanted cranial window during stroke (**Figure** **1**A). Our results show that astrocytes within the same field of view possessed decreased territories, and increased somas and soma‐territory ratio, on the first day post‐stroke (Figure 1B–H). Also, the fluorescent areas of numerous astrocytic processes were considerably reduced. Nevertheless, during the late acute phase of stroke, the somas became smaller, and soma‐territory ratios had decreased. There was a gradual restoration of astrocyte morphology to its pre‐stroke appearance. This suggests that the processes around the astrocytes had gradually recovered after the dysfunction caused by stroke.

To investigate the mechanisms driving these microdomain alterations in astrocytic processes, we integrated the single‐cell transcriptome sequencing data from peri‐infarct tissues^[^ ^26^ ^]^ and the astrocyte subcompartment proteomics data.^[^ ^27^ ^]^ Our results show 11 downregulated genes (*Sgip1*, *Nrxn1*, *Gpr158*, *Aak1*, *Rufy3*, *Alg2*, *Necap1*, *Abr*, *Atp8a1*, *Braf*, and *Hepecam*) and 3 upregulated genes (*Cct8*, *Tcp1* and *Msn*) expressing proteins specifically located in the astrocytic microdomain (Figure 1I–J; Figure S1, Supporting Information). We then verified the differential expression of these genes in tissue RNA samples from the peri‐infarct area (Figure 1K). Among the 14 genes, there were 12 genes showing significant changes which were considered markers of astrocyte microdomain dysfunction. Gene Ontology (GO) functional enrichment analysis of these 12 genes revealed that the top five significant GO terms were “vesicle‐mediated transport in synapse”, “regulation of vesicle‐mediated transport”, “response to calcium ions”, “endocytosis”, and “import into cell” (Figure 1L). The above functions in astrocytes were mediated by the Ca^2+^ transients,^[^ ^28^, ^29^, ^30^ ^]^ deciphering the complex Ca^2+^ transients in astrocytic microdomain underpinning is therefore of our request to understand the physiology of astrocytes.

### 2.2 The Astrocytic Microdomain Ca^2+^ Transients are Decreased in Awake Ischemic Stroke Mice

To characterize the changes in astrocytic Ca^2+^ activity after stroke, we utilized the *GfaABC_1_D* promoter to express the genetically encoded Ca^2+^ indicator GCaMP6f in cortical astrocytes. in vivo imaging of Ca^2+^ signaling in cortical astrocytes was performed using mTPM, which was used to record astrocytic Ca^2+^ activity via a cranial window. Mice in which the blood flow at the site of virus injection was reduced by ≈40–60% at 15 min after the performance of distal middle cerebral artery occlusion (dMCAO) were selected for subsequent experiments (Figure S2, Supporting Information).

**Figure 2:**
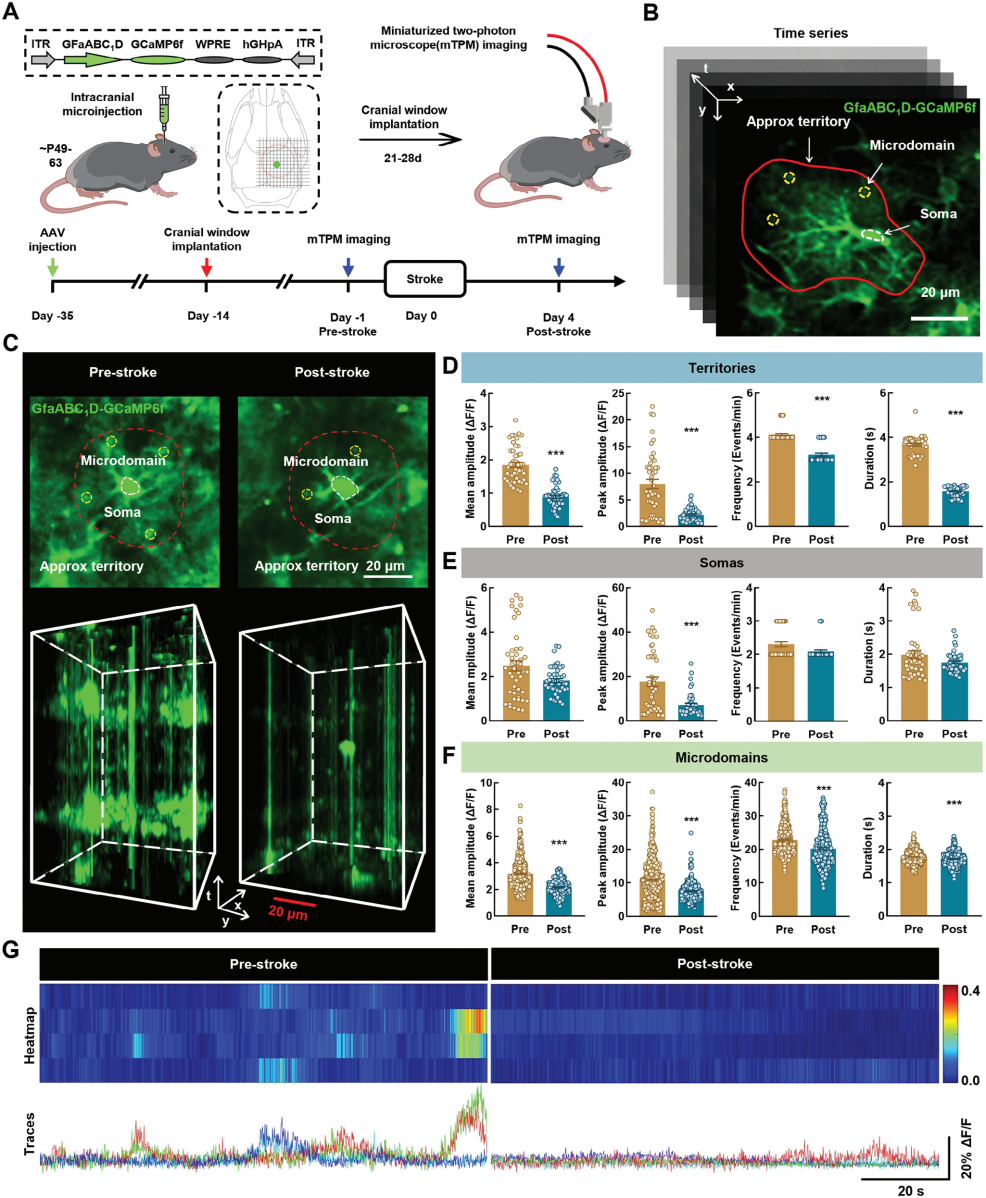
The astrocytic microdomain Ca^2+^ transients are decreased in awake ischemic stroke mice. A) Schematic of the approach for imaging of astrocyte calcium signals in vivo. Left: Cerebral cortex AAV injection for expressing GCaMP6f in astrocyte. Right: Astrocyte calcium signal imaging with mTPM. B) Representative time series images showing various regions of interest delineating the soma (dashed circle, white), microdomain (dashed circle, yellow) and approx territory (red outline). Scale bar, 20 µm. C) Representative images from Pre‐stroke and Post‐stroke mice showing spontaneous GCaMP6f activity in astrocytes. Red line depicts approximate territory. Scale bar, 20 µm. D–F) Quantification of GCaMP6f signal mean amplitude, peak amplitude, frequency, and duration derived from territory (D), soma (E), microdomain (F) from 3 mice. Pre = 48 territories, 45 somata, 461 microdomains; Post = 48 territories, 45 somata, 319 microdomains. Data are shown as mean ± SEM. ^***^ *P* < 0.001 versus the Pre group using the Mann‐Whitney *U* test. G) Representative microdomain traces and heat map of ΔF/F GCaMP6f signal. Scale bars, 20 s (horizontal) and 20% ΔF/F (vertical). mTPM: mini two‐photon microscope; Pre: pre‐stroke; Post: post‐stroke.

First, conscious mice with their heads secured in a fixed position were trained to acclimate to the imaging environment. Based on the distribution pattern of blood vessels in the field of view, we then obtained images of Ca^2+^ signaling in the same field of view before and after stroke. The mTPM was used to visualize Ca^2+^ signaling in cortical astrocytes. This signaling was classified as that occurring in the territory, soma, or microdomain according to the GECIquant algorithm^[^ ^12^ ^]^ (**Figure** **2**A,B). We observed that Ca^2+^ activity in astrocytes in the same field of view was decreased after stroke (Figure 2C). The amplitude, frequency, and duration of Ca^2+^ signaling in the astrocytic territory were also decreased (Figure 2D). Ca^2+^ activity occurring in the soma and microdomain were analyzed separately. The reduction in somatic Ca^2+^ transients was not significant, with only the peak amplitude showing a decrease, whereas all the parameters of the microdomain were decreased (Figure 2E–G). Taken together, our results indicate that the significant decrease observed in astrocytic Ca^2+^ transients was caused by the diminished microdomain Ca^2+^ transients, rather than that of somatic Ca^2+^ transients, in awake ischemic stroke mice.

### 2.3 Astrocytic Microdomain Ca^2+^ Transients are Attenuated During Free Behavior in Ischemic Stroke Mice with Impaired Sensorimotor Function

**Figure 3:**
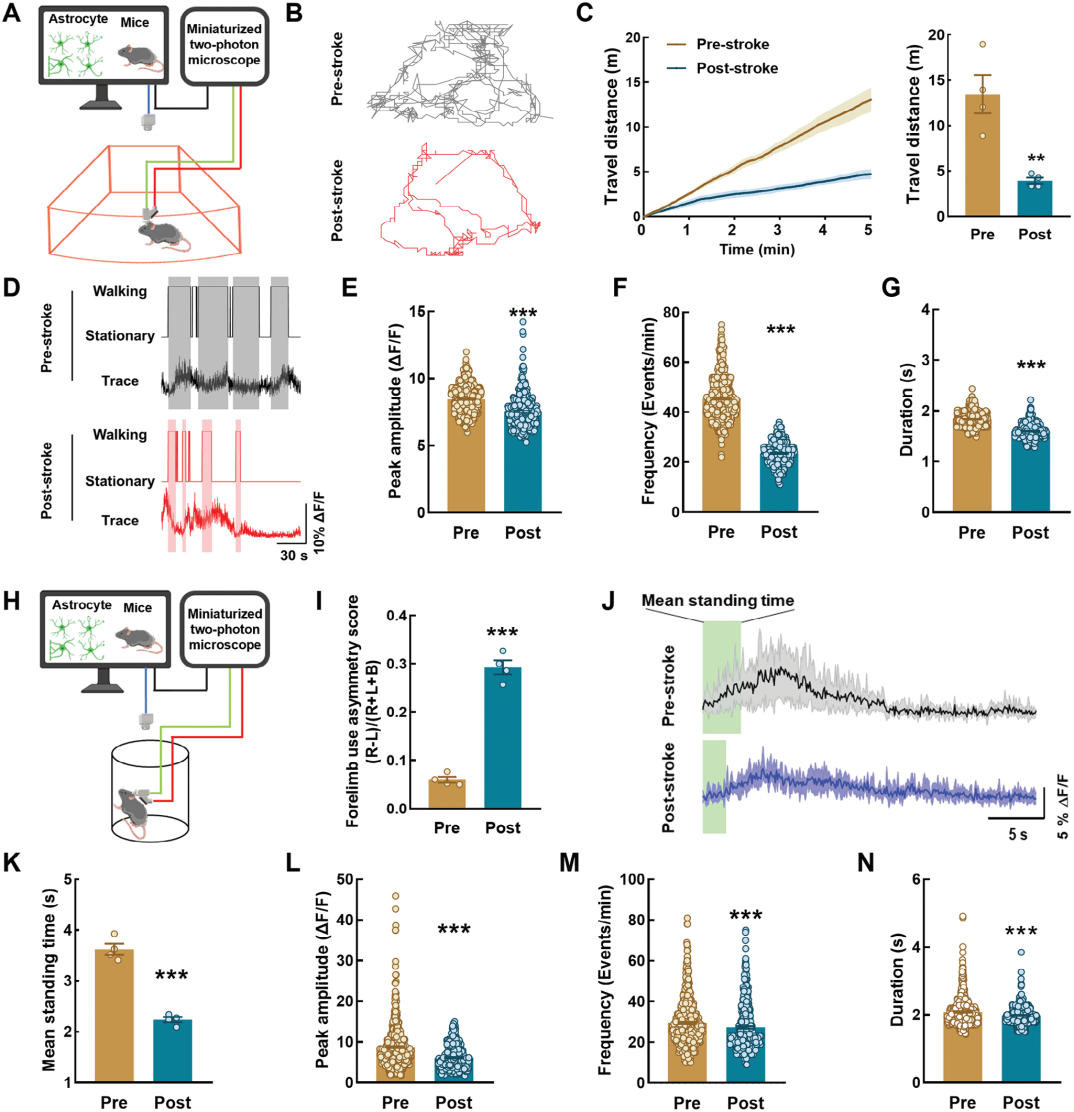
Astrocytic microdomain Ca^2+^ transients are attenuated during free behavior in ischemic stroke mice with impaired sensorimotor function. A) Schematic of the approach for imaging of astrocyte calcium signals in free moving mice with mTPM. B) Representative track traces of locomotor activity in free moving mice from Pre‐stroke and Post‐stroke mice. C) Travel distance in Pre‐stroke and Post‐stroke mice within 5 min. *n* = 4 mice. Data are shown as mean ± SEM. ^**^ *P *< 0.01 versus the Pre group using the Mann‐Whitney *U* test. D) Representative traces of microdomain Ca^2+^ in Pre and Post. Shaded areas indicate walking state. E–G) Quantification of microdomains GCaMP6f signal peak amplitude (E), frequency (F), and duration (G) derived from 552 microdomains from Pre‐stroke mice and 363 microdomains from Post‐stroke mice. Data are shown as mean ± SEM. ^***^ *P *< 0.001 versus the Pre group using the Mann‐Whitney *U* test. H) Schematic of the approach for imaging of astrocyte calcium signals in cylinder test with mTPM. I) Assessment of sensory and motor function. Data are shown as mean ± SEM. ^***^ *P *< 0.001 versus the Pre group using the Mann‐Whitney *U* test. J) Representative traces of microdomain Ca^2+^ in Pre and Post. Shaded areas indicate standing state. K) Graphs showing mean standing time in Pre and Post group, n = 4 mice. Data are shown as mean ± SEM. ^***^ *P *< 0.001 versus the Pre group using the Mann‐Whitney *U* test. L–N) Quantification of microdomains GCaMP6f signal peak amplitude (L), frequency (M), and duration (N) derived from 562 microdomains from Pre‐stroke mice and 364 microdomains from Post‐stroke mice. Data are shown as mean ± SEM. ^***^ *P *< 0.001 versus the Pre group using the Mann‐Whitney *U* test. Pre: pre‐stroke; Post: post‐stroke.

The distinct microdomain Ca^2+^ transients in astrocytes operate on different time scales within the neural circuit via the “triple synapse” and contribute to multiple behavioral outputs.^[^ ^16^, ^31^ ^]^ We further investigated the characteristics of astrocytic microdomain Ca^2+^ transients during behavioral deficits in freely behaving ischemic stroke mice. Astrocytic Ca^2+^ signaling and mouse behavior were simultaneously recorded (**Figure** **3**A). As described in the previous study,^[^ ^32^ ^]^ after mounting the mTPM headpiece onto the baseplate, the mice were initially provided with a 15‐min period to freely move about and adapt to the microscope in their cage. The trajectories of mouse movement were tracked using 5‐min video sessions, which revealed a significant reduction in locomotor distance after stroke compared with that at baseline (Figure 3B,C). We then combined Ca^2+^ imaging data with behavioral videos for analysis (Figure 3D), and found that the levels of astrocytic microdomain Ca^2+^ transients in walking mice were higher compared with those in a stationary state; this finding was also observed in ischemic stroke mice (Figure S3, Supporting Information). The levels of astrocytic microdomain Ca^2+^ transients in the walking state were also decreased following a stroke, as shown by analysis of the peak amplitude, frequency, and duration of GCaMP6f signals (Figure 3E–G).

In the cylinder test, mice showed a significant increase in the frequency of using the right forelimb to touch the cylinder wall after stroke (Figure 3H,I). Additionally, we combined the dynamic characterization of astrocytic microdomain Ca^2+^ activity with the behavioral state of mice during the first 30 s of testing (Figure 3J). During this period, the time of standing was significantly reduced in mice after stroke (Figure 3K); this was accompanied by a significant reduction in astrocytic microdomain Ca^2+^ transients during the 30‐s analysis (Figure 3L–N). The grid‐walking test showed a concurrent increase in the rate of foot faults, and a decrease in astrocytic microdomain Ca^2+^ transients, during the 30‐s walk (Figure S4, Supporting Information). These observations indicate a reduction in astrocytic microdomain Ca^2+^ transients during unconstrained behavior in mice with compromised sensorimotor function, highlighting potential implications for neuroscientific understanding.

### 2.4 The Mitochondrial Ca^2+^ Transients in Astrocytic Microdomains are Diminished After Stroke

**Figure 4:**
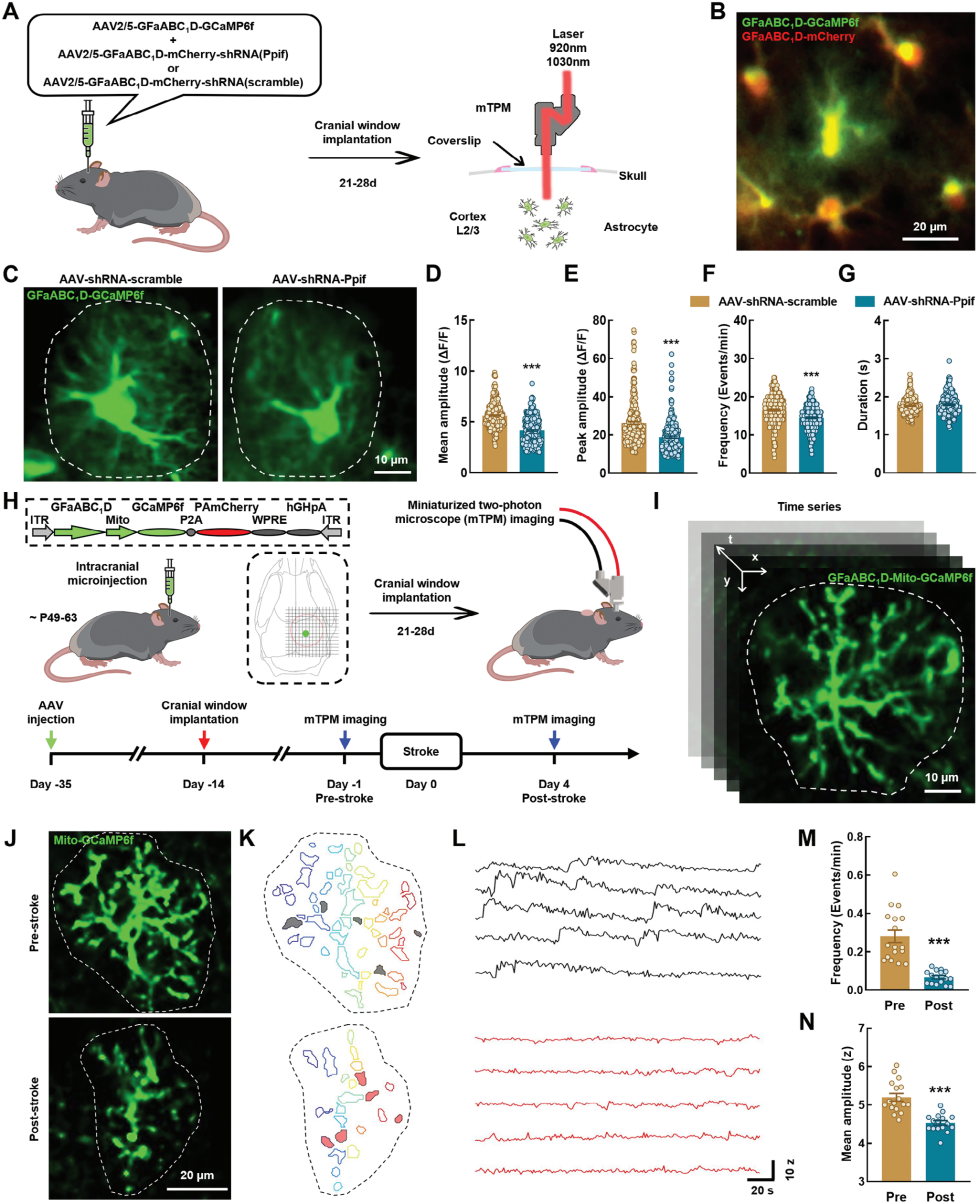
The mitochondrial Ca^2+^ transients in astrocytic microdomains are diminished after stroke. A) Schematic of AAVs administration and two‐channel miniaturized two‐photon microscope imaging. B) Representative image show dual channel imaging. Scale bar, 20 µm. C) Representative images from healthy mice showing spontaneous GCaMP6f activity in astrocytes. Scale bar, 10 µm. D–G) Quantification of microdomains GCaMP6f signal mean amplitude (D), peak amplitude (E), frequency (F), and duration (G) derived from AAV‐shRNA‐scramble group (302 microdomains, 3 mice) and AAV‐shRNA‐Ppif group (252 microdomains, 3 mice). Data are shown as mean ± SEM. ^***^ *P *< 0.001 versus the AAV‐ shRNA‐scramble group using the Mann‐Whitney *U* test. H) Schematic of the approach for imaging of astrocyte mitochondrial calcium signals in vivo. Left: Cerebral cortex AAVs injection for expressing mitoGCaMP6f in astrocyte. Right: Astrocyte mitochondrial calcium signal imaging with mTPM. I) Representative time series images showing astrocyte mitochondrial calcium signals. Scale bar, 10 µm. J) Median intensity projection image of 3000 frames from one astrocyte in one mouse from Pre‐stroke and Post‐stroke group. Dashed lines indicate cell border. Scale bar, 20 µm. K) Map of microdomains recorded in (J). L) Intensity versus time traces for five microdomains (corresponding to colors in K) showing characteristics of Ca^2+^ transients in different groups. M–N) Graphs showing events/domain and mean amplitude in Pre‐stroke (17 cells, 4 mice) and Post‐stroke (16 cells, 4 mice) mice. Data are shown as mean ± SEM. ^***^ *P *< 0.001 versus the Pre group using the Mann‐Whitney *U* test. mTPM: mini two‐photon microscope; Pre: pre‐stroke; Post: post‐stroke.

High‐resolution serial electron microscopy has shown that mitochondria in the astrocytic processes are distributed close to the synapses,^[^ ^33^ ^]^ and that Ca^2+^ efflux from the mitochondria contributes to astrocytic microdomain Ca^2+^ transients.^[^ ^20^ ^]^ To elucidate the role of the mitochondria in the generation of astrocytic microdomain Ca^2+^ transients, we specifically inhibited the activity of the astrocyte mitochondrial permeability transition pore (mPTP), which controls mitochondrial Ca^2+^ efflux. An AAV targeting astrocytes was used to knock down the expression of *Ppif*, an essential element of mPTP (**Figure** **4**A,B). Our results show that the levels of astrocytic microdomain Ca^2+^ transients were dramatically decreased after the knockdown of *Ppif* expression (Figure 4C–G). These results show that the generation of microdomain Ca^2+^ transients was considerably influenced by that of mitochondrial Ca^2+^ transients.

We further investigated the changes of mitochondrial Ca^2+^ transients in the astrocytic microdomain. GCaMP6f was specifically expressed in the astrocytic mitochondria under the control of *GfaABC_1_D* promoter, and astrocytic microdomain mitochondrial Ca^2+^ signals were imaged using mTPM (Figure 4H,I). Our results show that the astrocytic microdomain mitochondrial Ca^2+^ transients were significantly reduced on day 4 after stroke compared with their levels at baseline (Figure 4J). Next, we employed a semi‐automatic CaSCaDE algorithm to analyze the frequency and amplitude of the astrocytic microdomain mitochondrial Ca^2+^ transients.^[^ ^20^ ^]^ The microdomain map and representative traces show that the microdomain mitochondrial Ca^2+^ transients were decreased in awake ischemic stroke mice (Figure 4K,L). Moreover, the number of events per domain and average event amplitude were also decreased in these mice after stroke (Figure 4M,N). Collectively, these findings show that the astrocytic microdomain mitochondrial Ca^2+^ transients were reduced in these mice after a stroke.

### 2.5 DDX1 Binding with circSCMH1 Promotes the Formation of MARVs

**Figure 5:**
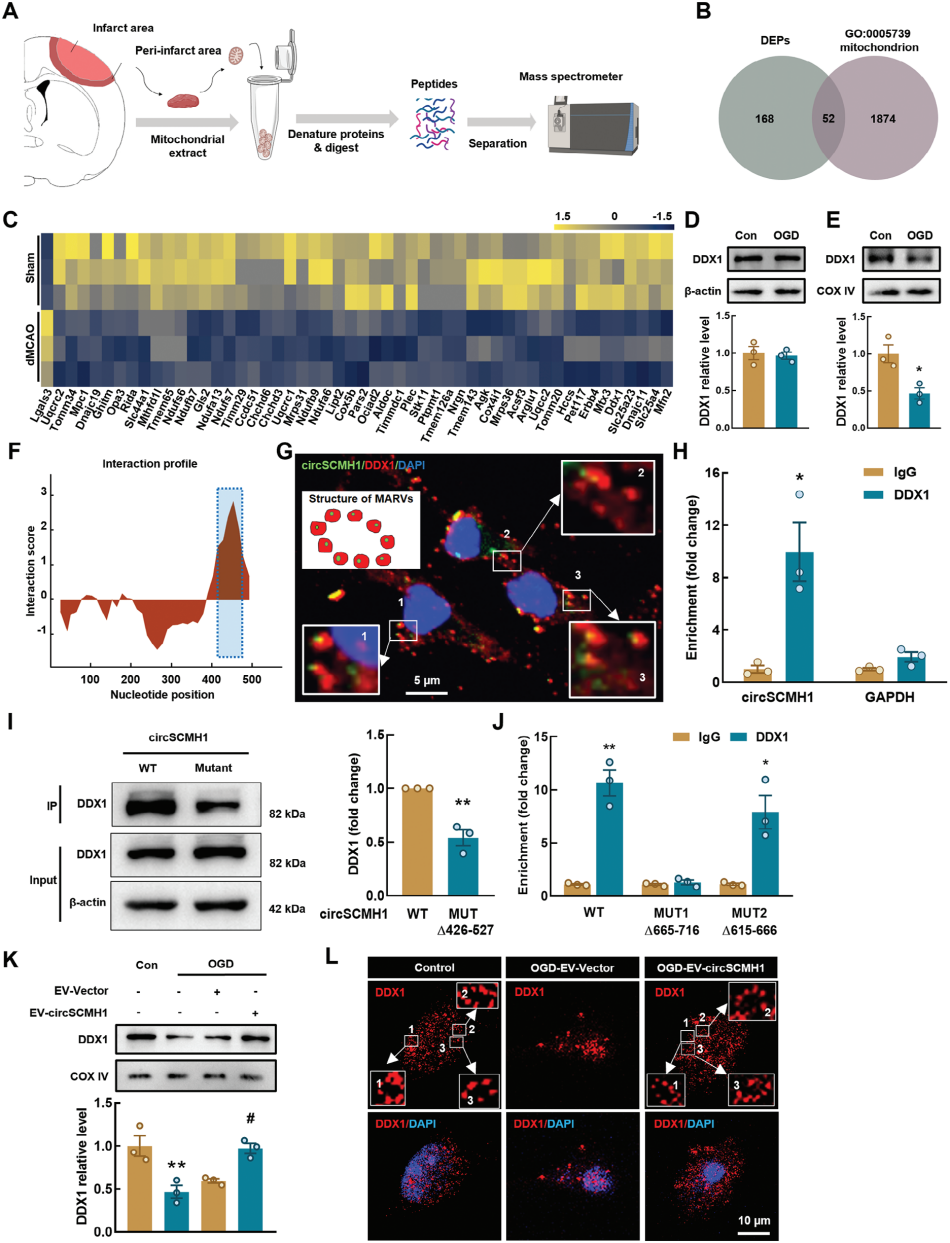
DDX1 binding with circSCMH1 promotes the formation of MARVs. A) Schematic illustration of mitochondrial protein mass spectrometry from the peri‐infarct area. B) Venn diagram showing numbers of proteins with significant changes in expression (DEPs: fold change > 1.1 or < 0.5, *P *< 0.05). DEPs: differentially expressed proteins. C) The heatmap shows the different expression of the 52 proteins in (B) proteins expression levels are standardized and displayed as gradient colors from higher (olive yellow) to lower (misty blue). D) Western blot analysis showing that the DDX1 level does not altered in OGD model (6h) of primary mouse astrocytes. Three representative immunoblots are presented. Data are shown as mean ± SEM. E) Western blot analysis showing that DDX1 is decreased in astrocytic mitochondrion (OGD, 6 h) compared with the control. Three representative immunoblots are presented. Data are shown as mean ± SEM. ^*^ *P *< 0.05 versus the control group using the Student's *t* test. F) Prediction of circSCMH1‐DDX1 interaction by catRAPID algorithm. G) Colocalization of DDX1 and circSCMH1 in primary astrocytes. Scale bar: 5 µm. H) Interaction between circSCMH1 and DDX1 is validated by RNA immunoprecipitation (RIP) in primary astrocytes. Data are presented as mean ± SEM of 3 independent experiments. ^*^ *P *< 0.05 versus the IgG group using the Student's *t* test. I) Western blot analysis of DDX1 expression in lysates of primary astrocytes with circSCMH1 or mutated circSCMH1 (Δ426–477) overexpression following biotinylated circSCMH1 probe pull‐down assay. Data are shown as mean ± SEM. ^**^ *P *< 0.01 versus the WT group using the Student's *t* test. J) Relative enrichment of endogenous circSCMH1 in mutant DDX1 RIP as measured by qPCR. Data are presented as mean ± SEM of 3 independent experiments. ^*^ *P *< 0.05, ^**^ *P *< 0.01 versus IgG group using the Student's *t* test. K) Representative western blots of DDX1 expression in mitochondrion of primary astrocytes with EV‐circSCMH1 transfection at OGD condition. Three representative immunoblots are presented. Data are shown as mean ± SEM. ^**^ *P *< 0.01 versus the control group; ^#^ *P *< 0.05 versus the OGD‐EV‐Vector group using one‐way ANOVA followed by the Holm–Sidak post hoc multiple comparisons test. L) Representative images of astrocyte immunostaining for DDX1 which participates in the formation of MARVs. Scale bars: 10 µm. OGD: oxygen‐glucose deprivation, WT: Wild type, MUT: Mutant.

To investigate the mechanisms involved in the regulation of mitochondrial Ca^2+^ transients, we extracted the mitochondria from the peri‐infarct region of ischemic stroke mice and analyzed the extracts using protein mass spectrometry (**Figure** **5**A). In the stroke group, we identified a total of 220 differentially expressed proteins, of which 28 showed an upregulated, and 192 showed a downregulated expression (Table S1, Supporting Information). Subsequently, we performed a functional annotation using Gene Ontology (GO) and identified a subset of 52 proteins involved in mitochondrial function (GO:0005739) (Figure 5B,C). We then focused on the downregulated expression of the DDX1 protein, which is an essential component of the MARVs that regulate mitochondrial Ca^2+^ distribution.^[^ ^21^ ^]^ The expression of DDX1 was examined in an in vitro oxygen‐glucose deprivation (OGD) model of ischemic stroke. Interestingly, the total protein level of DDX1 in astrocytes was not altered (Figure 5D); however, the level of DDX1 in astrocytic mitochondria was reduced significantly in OGD (Figure 5E). These findings suggest that the transport of DDX1 to the mitochondria of astrocytes was impaired after ischemia, potentially contributing to alterations in microdomain Ca^2+^ transients.

Electron microscopy revealed RNA cores in the ring‐like structures of MARVs, similar in appearance to the structure of circular RNA. Our previous study has shown that four circRNAs (circHECTD1, circTLK1, circDLGAP4, and circSCMH1) are closely associated with stroke.^[^ ^34^, ^35^, ^36^, ^37^ ^]^ We then used the catRAPID algorithm to predict the interaction propensity between DDX1 and these circRNAs. Among these four circRNAs, circSCMH1 had the highest propensity to bind to DDX1 (Figure 5F; Table S2, Supporting Information). Fluorescence in‐situ hybridization (FISH) combined with immunofluorescence labeling was used to verify that DDX1 bound with circSCMH1, resulting in the formation of MARVs in primary astrocytes (Figure 5G). This interaction was confirmed experimentally using RNA immunoprecipitation (RIP) (Figure 5H).

Moreover, the binding sites of circSCMH1 and DDX1 were predicted through bioinformatics analysis using catRAPID, which identified that the 426–527 region of circSCMH1 has a strong binding capacity with the 665–716 and 615–666 regions of DDX1. Consequently, we first constructed a circSCMH1 mutant plasmid (Δ426–527), wherein we deleted the binding sequence of circSCMH1 (426–527) that interacts with DDX1. By transducing either the WT or mutant circSCMH1 plasmid and performing pull‐down analysis, we found that the unmodified circSCMH1 exhibited significantly stronger binding with DDX1 in primary mouse astrocytes compared to the circSCMH1 mutant plasmid (Δ426–527) (Figure 5I). Given that two regions of DDX1 (residues 665–716 and 615–666) were predicted to have a high interaction capacity with circSCMH1, we generated DDX1 variants lacking either residues 665–716 or 615–666 to explore this interaction further. RNA‐binding assays revealed a decreased level of interaction between circSCMH1 and the Δ665‐716, but not the Δ615‐666, DDX1 variant compared to the wild‐type DDX1 (DDX1‐WT) (Figure 5J). These findings suggest that the binding interaction between circSCMH1 and DDX1 necessitates the presence of residues 665–716 in DDX1.

Additionally, we observed that EV delivery of circSCMH1 effectively counteracted the decrease in DDX1 mitochondrial localization induced by OGD (Figure 5K). Notably, upregulation in circSCMH1 expression significantly rescued the dysfunction in MARVs subjected to OGD, as shown by immunofluorescence labeling (Figure 5L). Specific knockdown of DDX1 expression in astrocytes substantially attenuated the effect of circSCMH1 on microdomain mitochondrial morphology, as evidenced by transmission electron microscopy (TEM) (Figure S5, Supporting Information). Together, these results show that DDX1 combined with circSCMH1 to form MARVs, promote the transport of Ca^2+^ into the mitochondria, and affect the generation of microdomain Ca^2+^ transients.

### 2.6 Interaction Between circSCMH1 and DDX1 Ameliorates Astrocytic Microdomain Ca^2+^ Transients after Stroke

Next, we investigated the effects of MARVs consisting of DDX1 and circSCMH1 on microdomain Ca^2+^ transients in astrocytes. For this, we first evaluated the effects of circSCMH1 on astrocytic microdomain Ca^2+^ transients by delivering circSCMH1 via EV to the brain tissue of ischemic stroke mice. Using genetic encoding, we assessed the astrocytic Ca^2+^ transients and quantified the microdomain GCaMP6f signal peak amplitude, frequency, and duration (Figure S6, Supporting Information). Our results show that infusion of EV‐circSCMH1 significantly reversed the decrease in microdomain Ca^2+^ transients caused by stroke in astrocytes.

**Figure 6:**
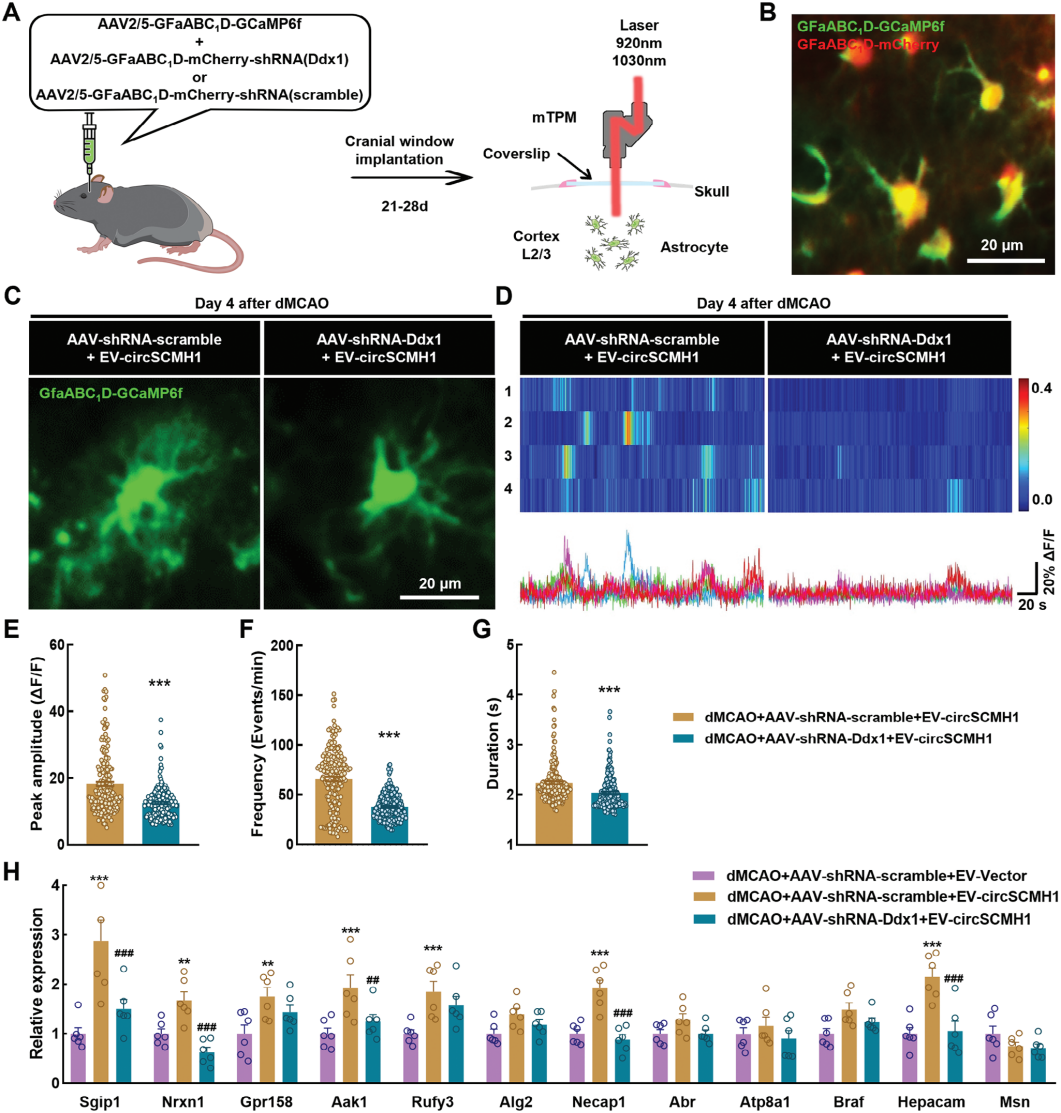
Interaction between circSCMH1 and DDX1 ameliorates astrocytic microdomain Ca^2+^ transients after stroke. A) Schematic of AAVs administration and two‐channel miniaturized two‐photon microscope imaging. B) Representative image show dual channel imaging. Scale bar, 20 µm. C) Representative images from stroke mice showing spontaneous GCaMP6f activity in astrocytes. Scale bar, 20 µm. D) Representative microdomain traces and heat map of GCaMP6f signal from Post‐stroke mice. Scale bars, 20 s (horizontal) and 20% ΔF/F (vertical). E–G) Quantification of microdomains GCaMP6f signal peak amplitude (E), frequency (F), and duration (G) in dMCAO + EV‐circSCMH1 + AAV‐shRNA‐scramble (190 microdomains, 3 mice) and dMCAO+ EV‐circSCMH1 + AAV‐shRNA‐Ddx1 (203 microdomains, 3 mice) group. Data are shown as mean ± SEM. ^*^ *P *< 0.05, ^***^ *P *< 0.001 versus the dMCAO + EV‐circSCMH1 + AAV‐shRNA‐scramble group using the Student's *t* test. H) qRT‐PCR validation of 12 gene differentially expressed in peri‐infarct area. *n* = 6 mice per group. Data are shown as mean ± SEM. ^**^ *P *< 0.01, ^***^ *P *< 0.001 versus the dMCAO + EV‐Vector + AAV‐shRNA‐scramble group; ^##^ *P *< 0.01, ^###^ *P *< 0.001 versus the dMCAO + EV‐circSCMH1 + AAV‐shRNA‐scramble group using one‐way ANOVA followed by Holm‐Sidak post hoc multiple comparisons test.

Next, a mixture of AAV‐GfaABC_1_D‐GCaMP6f and AAV‐GfaABC_1_D‐mcherry‐shRNA (Ddx1) or AAV‐GfaABC_1_D‐mcherry‐shRNA (scramble) was stereotaxically injected into the mouse cortex. We then examined the changes of microdomain Ca^2+^ transients in the astrocytes of DDX1 knockdown mice following treatment with EV‐circSCMH1 (**Figure** **6**A). Two‐photon dual channel imaging confirmed that the two types of viruses had successfully targeted a single cell simultaneously (Figure 6B). First, we analyzed the effect of specific DDX1 knockdown in astrocytes on microdomain Ca^2+^ transients. Our results show that the astrocytic microdomain Ca^2+^ transients were decreased significantly in DDX1 knockdown mice (Figure S7, Supporting Information). Subsequently, we used the administration of EV‐circSCMH1 to compare astrocytic microdomain Ca^2+^ transients in control and DDX1 knockdown mice after stroke (Figure 6C,D). Interestingly, the peak amplitude, frequency, and duration of astrocytic microdomain Ca^2+^ transients were all reduced in DDX1 knockdown mice (Figure 6E–G). The results of qPCR showed that the expression levels of most microdomain genes (*Sgip1*, *Nrxn1*, *Gpr158*, *Aak1*, *Rufy3*, *Necap1*, and *Hepacam*) were increased after the stroke via administration of EV‐circSCMH1; only 5 microdomain genes (*Sgip1*, *Nrxn1*, *Aak1*, *Necap1*, and *Hepacam*) showed downregulated expression levels after circSCMH1‐mediated intervention in DDX1 knockdown mice (Figure 6H). These results suggest that DDX1 and circSCMH1, two components of MARVs, were both required to restore the levels of astrocyte microdomain Ca^2+^ transients after stroke.

### 2.7 Neuronal Ca^2+^ Transients are Amplified by circSCMH1‐DDX1 Interaction in Astrocytes After Stroke

Cortical astrocytes play crucial roles in integrating local sensory information and influencing the behavioral state by regulating the synaptic strength using alterations in free intracellular Ca^2+^ levels at peri‐synaptic compartments.^[^ ^17^, ^38^ ^]^ Therefore, we next investigated whether Event I (circSCMH1 promotes astrocytic microdomain Ca^2+^ transients) was involved in Event II (circSCMH1 participates in post‐stroke motor functional recovery) in order to determine the effects of EV‐circSCMH1 administration on the functional recovery of neurons damaged by stroke.

First, we used the *hSyn* promoter to express GCaMP6s in cortical neurons. Then, we assessed the effects of stroke and circSCMH1 expression on neuronal Ca^2+^ transients (Figure S8A—C, Supporting Information). Our results show that a significant reduction in neuronal Ca^2+^ transients occurred in the awake mice after stroke. However, treatment using EV‐circSCMH1 reversed the decrease in neuronal Ca^2+^ transients (Figure S8D, Supporting Information). Stroke in the motor cortex leads to a scattered organization of motor maps, indicating a reduction in local spatial correlation.^[^ ^39^ ^]^ Using the Pearson correlation and network analysis, we designated neurons in the field of view into several ensembles based on the similarity of their activities. Our results show that the number of neuronal ensembles was increased after stroke, while the number of ensembles in the EV‐circSCMH1 group was lower than that in the EV‐Vector group (Figure S8E, Supporting Information). These findings indicate that treatment with circSCMH1 significantly enhanced the synergy of neuronal function in awake ischemic stroke mice.

**Figure 7:**
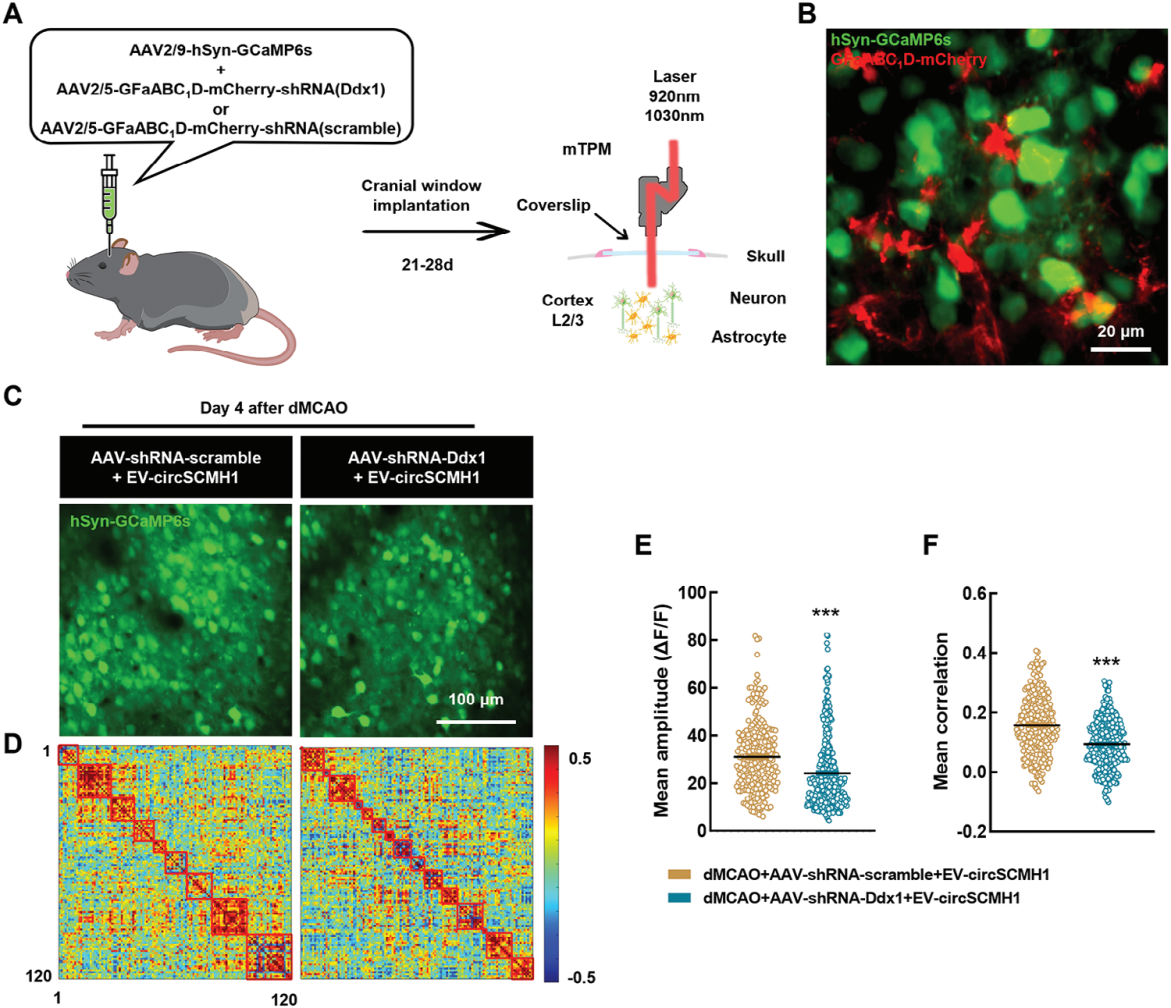
Neuronal Ca^2+^ transients are amplified by circSCMH1‐DDX1 interaction in astrocytes after stroke. A) Schematic of AAVs administration and two‐channel miniaturized two‐photon microscope imaging. B) Representative image show dual channel imaging. Scale bar, 20 µm. C) Representative images from stroke mice showing spontaneous GCaMP6s activity in neurons. Scale bar, 100 µm. D) Map of correlation coefficient matrix among 120 neurons. E,F) Quantification of microdomains GCaMP6s signal mean amplitude (E) and mean correlation (F) in dMCAO + EV‐circSCMH1 + AAV‐shRNA‐scramble (253 neurons, 3 mice) and dMCAO+ EV‐circSCMH1 + AAV‐shRNA‐Ddx1 (317 neurons, 3 mice) group. Data are shown as mean ± SEM. ^***^ *P *< 0.001 versus the dMCAO + EV‐circSCMH1 + AAV‐shRNA‐scramble group using the Mann‐Whitney *U* test.

We also investigated the effect of MARVs on neuronal Ca^2+^ transients by using AAV to knock down the expression of astrocyte DDX1 (**Figure** **7**A). Imaging using two‐photon dual channel microscopy confirmed that GCaMP6s was specifically expressed on neurons, and that mCherry was specifically expressed on astrocytes, in the same field of view (Figure 7B). We delivered circSCMH1 via the administration of EV‐circSCMH1 on day 1 post‐stroke. Our results show that the levels of neuronal Ca^2+^ transients were significantly decreased in mice with astrocyte‐specific DDX1 knockdown after the administration of EV‐circSCMH1 under stroke conditions (Figure 7C–E). The synergy of neuronal function in DDX1‐knockdown mice was also significantly weakened following stroke (Figure 7F). Together, these results indicate that the enhanced astrocytic microdomain Ca^2+^ transients by the binding of circSCMH1‐DDX1 promoted the recovery of neuronal function after stroke.

### 2.8 EV Delivery of circSCMH1 Improves Motor Function Recovery After Stroke via Astrocytic DDX1

**Figure 8:**
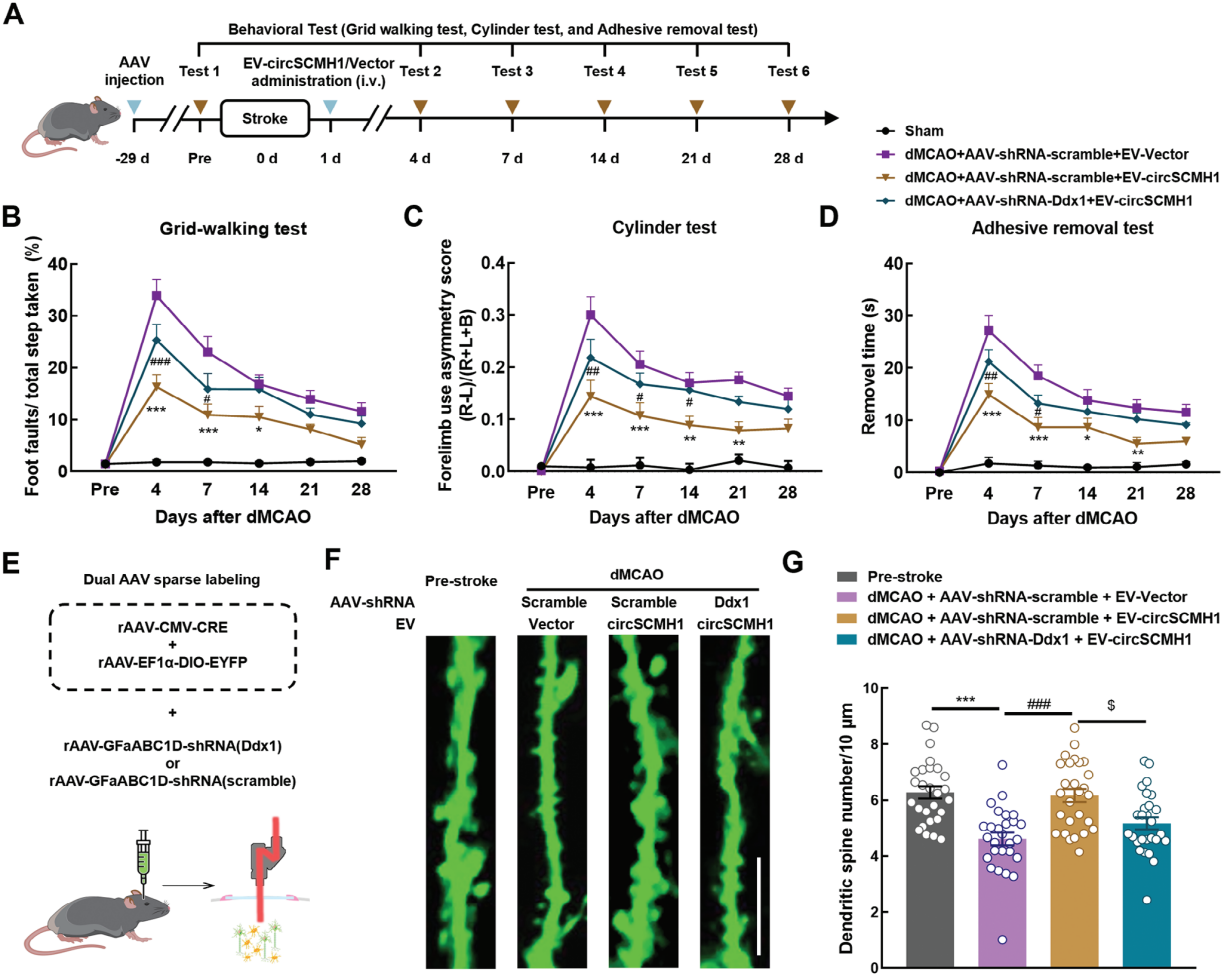
EV delivery of circSCMH1 improves motor function recovery after stroke via astrocytic DDX1. A) Schematic of the experimental procedure and behavioural studies. B–D) DDX1 knock down inhibited the improvement of behavioral recovery caused by circSCMH1 intervention by the grid‐walking test (B), cylinder test (C), and adhesive removal test (D). L indicates left forepaw in cylinder test; R, right forepaw in cylinder test; B, both forepaws in cylinder test. *n* = 12 mice per group. ^*^ *P *< 0.05, ^**^ *P *< 0.01, ^***^ *P *< 0.001 versus the dMCAO + AAV‐shRNA‐scramble + EV‐Vector group; ^#^ *P *< 0.05, ^##^ *P *< 0.01, ^###^ *P *< 0.001 versus the dMCAO + AAV‐shRNA‐scramble + EV‐circSCMH1 group; two‐way repeated‐measures ANOVA followed by Holm–Sidak post hoc multiple comparison test. E) Strategy for labeling the neurons sparsely and knockdown of Ddx1 specifically in astrocytes to study the dendritic spines through in vivo two‐photon imaging during stroke. F) Representative images of two‐photon spine imaging from mice pre‐stroke and 4 days after the dMCAO model. Scale bar, 10 µm. G) Quantitation of dendritic spine numbers per 10 µm. *n* = 27 dendrites from 3 mice, ^***^ *P *< 0.001 versus the Pre‐stroke group, ^###^ *P *< 0.001 versus the dMCAO + AAV‐shRNA‐scramble + EV‐Vector group, and ^$^ *P *< 0.05 versus dMCAO + AAV‐shRNA‐scramble + EV‐circSCMH1 group using one‐way ANOVA followed by the Holm‐Sidak test.

Next, we further investigated whether increasing the levels of astrocytic microdomain Ca^2+^ transients using EV‐circSCMH1 improved the recovery of sensorimotor function in dMCAO mice. For this, we administered AAV‐GfaABC_1_D‐shRNA‐Ddx1 or AAV‐GfaABC_1_D‐shRNA‐scramble, and evaluated motor function recovery after dMCAO surgery and EV‐circSCMH1 administration in these mice (**Figure** **8**A). Mice injected with EV‐circSCMH1 showed improved performance in the grid‐walking test with reduced foot faults at day 4, 7, and 14 post‐dMCAO surgery compared with the performance of the mice treated with EV‐Vector. However, these effects were significantly attenuated after astrocyte‐specific DDX1 knockdown (Figure 8B). Similar results were obtained using the cylinder test, in which mice in the EV‐circSCMH1 + AAV‐shRNA‐Ddx1 group showed increased bias at day 4, 7, and 14 post‐dMCAO surgery compared with that of the EV‐circSCMH1 + AAV‐shRNA‐scramble mice (Figure 8C). Likewise, in the adhesive removal test, the DDX1 knockdown mice required more removal time than control mice after stroke (Figure 8D). These findings indicated that astrocyte‐specific DDX1 mediated the circSCMH1‐induced functional recovery after stroke.

Additionally, through 2,3,5‐triphenyltetrazolium chloride (TTC) staining analysis, we confirmed that circSCMH1 significantly decreased the cerebral infarct volume at day 4 after stroke. This protective effect was significantly inhibited by knocking down of DDX1 (Figure S9A,B, Supporting Information). NeuN staining in the peri‐infarct region revealed that circSCMH1 markedly hindered post‐stroke neuronal loss, which was mitigated by DDX1 knockdown (Figure S9C,D, Supporting Information). Consistent with the analysis of neuronal loss, EV‐circSCMH1 treatment significantly enhanced the spine numbers in the peri‐infarct cortex of mice at day 4 after stroke, and DDX1 knockdown attenuated the beneficial effects of EV‐circSCMH1 treatment (Figure 8E–G). Furthermore, using fluorescent labeling of neuronal calcium activity and in vivo two‐photon imaging, we found that enhancing astrocytic microdomain calcium activity through EV‐circSCMH1 treatment significantly increased the amplitude and frequency of Ca^2+^ transients in neuronal synapses. This effect was inhibited by DDX1 knockdown in dMCAO mice (Figure S10, Supporting Information). These results indicated that astrocyte‐specific DDX1 mediated the circSCMH1‐induced repair of neuronal function after stroke.

## 3 Discussion

Our present study provides a comprehensive and dynamic analysis of astrocytic microdomain Ca^2+^ transients and their correlation with behavioral deficits in freely‐moving ischemic stroke mice. We have also identified a novel mechanism driven by mitochondrial impairment, which led to a reduction in microdomain Ca^2+^ transients in astrocytes after stroke. Specifically, we found that mitochondrial Ca^2+^ transients were regulated by MARVs. Within this frame, DDX1 was identified as a crucial factor in the formation of ring‐like MARVs. By interacting with circSCMH1, DDX1 participated in the formation of MARVs, thereby enhancing the transport of Ca^2+^ to the mitochondria, and ultimately promoting the generation of microdomain Ca^2+^ transients after stroke. These findings suggest that an astrocytic microdomain Ca^2+^‐based mechanism was underlying the observed behavioral changes in mice after stroke.

The lack of successful translation from animal studies of stroke to clinical trials has resulted in considerable skepticism regarding the neuroprotection hypothesis.^[^ ^40^ ^]^ Previous studies have predominantly focused on rescuing neurons directly, neglecting the role of other cells that are crucial for neuronal preservation. In recent years, several studies have highlighted the effects of astrocytes on neuronal dysfunction, and synaptic damage and recovery, which are crucial for the recovery of sensorimotor functions after stroke.^[^ ^41^, ^42^ ^]^ A functional synapse involves neuronal elements and astrocytic processes that respond to neurotransmitter release and influence synaptic activity via feedback.^[^ ^43^ ^]^ However, whether post‐stroke abnormal activity in astrocytic processes is involved in behavioral changes, and the potential mechanisms underlying this activity, remain poorly understood. Microdomain Ca^2+^ transients in astrocytic processes have been implicated in the local control of neural circuits in various species.^[^ ^14^, ^44^, ^45^ ^]^ In the present study, we show the effects of intervening in the generation of astrocytic microdomain Ca^2+^ transients on the recovery of sensorimotor functions in ischemic stroke mice. Our results strongly suggest that detailed investigations into how abnormal astrocytic microdomain Ca^2+^ transients are implicated in behavioral deficits post‐stroke will provide important new insights into the function of astrocytic microdomain Ca^2+^ transients.

Astrocytes respond to disruptions in the microenvironment homeostasis and regulate synaptic function via intracellular Ca^2+^ changes.^[^ ^17^ ^]^ There are two major types of Ca^2+^ signaling events in astrocytes: whole‐cell fluctuations and microdomain Ca^2+^ transients.^[^ ^46^ ^]^ Due to limitations in imaging techniques and algorithms, studies on astrocytic Ca^2+^ transients after stroke have been primarily focused on peri‐infarction depolarization (PID). PID is a whole‐cell fluctuation that contributes to secondary infarct growth and negatively affects stroke outcome.^[^ ^47^, ^48^ ^]^ This type of astrocytic Ca^2+^ signaling is increased at 1–2 h after a photothrombotic stroke in both the ischemic core and the penumbra.^[^ ^49^ ^]^ The PID‐related astrocytic Ca^2+^ signals are contributed by IP_3_R_2_‐gated Ca^2+^ release from the endoplasmic reticulum and Ca^2+^ influx through the TRPV4 channels from the extracellular medium. However, one study found that behavioral processes, such as motor and sensory function, remain unaffected by the lack of astrocyte IP_3_R_2_‐mediated Ca^2+^ signals in astrocytic IP_3_R_2_‐conditional knockout mice.^[^ ^50^ ^]^ Therefore, we employed the recent technological advancements to decipher the characteristics of astrocytic microdomain Ca^2+^ transients after stroke. Using a two‐photon imaging platform, we monitored dynamically and in real‐time the changes in astrocyte microdomain Ca^2+^ transients after stroke, and identified characteristics that were, to a significant extent, functionally distinct from whole‐cell fluctuations. Simultaneously, the relationship between astrocytic microdomain Ca^2+^ transients and post‐stroke behavioral recovery has further emphasized the practical significance of studying the mechanisms underlying these processes.

Unlike whole‐cell fluctuations, a subset of microdomain Ca^2+^ transients is associated with the mitochondria.^[^ ^20^, ^51^ ^]^ Mitochondria are abundant in astrocytic processes, showing a density comparable to that found in nerve terminals, in which there exists a high metabolic demand.^[^ ^20^ ^]^ Microdomain Ca^2+^ transients in astrocytes showed spatial correlation with the mitochondria. Inhibition of the mPTP function significantly reduced the microdomain Ca^2+^ transients. Moreover, a previous study has also demonstrated the effects of extracellular Ca^2+^ influx on microdomain Ca^2+^ transients in astrocytes, suggesting the presence of a mechanism that regulates microdomain Ca^2+^ transients in astrocytes independent of the mitochondria.^[^ ^52^ ^]^ In the present study, we have unveiled a mitochondria‐dependent mechanism that regulated the astrocytic microdomain Ca^2+^ transients after stroke. However, we could not rule out the effects of Ca^2+^ release from the endoplasmic reticulum and influx of extracellular Ca^2+^ on astrocytic microdomain Ca^2+^ transients. These factors necessitate further in‐depth exploration of the role, and mechanisms underlying the activity, of astrocytic microdomain Ca^2+^ transients.

The molecular mechanisms regulating astrocytic microdomain Ca^2+^ transients remain largely unexplored. We initially focused on investigating the molecules involved in the regulation of mitochondria‐mediated astrocytic microdomain Ca^2+^ transients. The DDX1/RNAs/Ca^2+^‐containing MARVs enable the transfer of Ca^2+^ from MARVs to the mitochondria following an ion gradient.^[^ ^21^ ^]^ In the present study, we found that DDX1 in astrocytes interacted with circSCMH1 to envelop the Ca^2+^‐containing MARVs, thereby modulating the mitochondrial Ca^2+^ transients and influencing the microdomain Ca^2+^ transients. We confirmed the conservation of this mechanism in different cells, and identified DDX1 as one of the main components involved in the interaction within MARVs. Interestingly, mitochondrial Ca^2+^ transients in the distal astrocytic microdomain showed increased fragmentation, or even disappeared, after stroke. We speculate that mitochondrial fission may have been caused by the inability of MARVs to deliver Ca^2+^ to the mitochondria. Further investigations are required to investigate the specific mechanisms involved in astrocyte‐mediated regulation of microdomain Ca^2+^ transients, and the effects of these mechanisms on synaptic function and neural circuitry. Our study indicated that circSCMH1‐DDX1 interaction in astrocytes affected neuronal Ca^2+^ transients after stroke. Even so, we couldn't rule out the possibility that other mechanisms underlying the direct regulation of Ca^2+^ transients in neurons. Understanding these molecular processes may open up new avenues in the development of targeted interventions aimed at enhancing post‐stroke recovery and neuroprotection.

Overall, the results obtained in the present study will contribute to the growing body of knowledge of astrocytic microdomain Ca^2+^ signaling and its relevance in neurologic disorders. Our findings provide important insights into the mechanisms involved in stroke, and establish a groundwork for subsequent studies seeking to unravel the complexities of astrocytic involvement in brain function and pathology.

## 4 Experimental Section

### Animals

Adult male C57BL/6J mice (6‐8 weeks) were purchased from the GemPharmatech (Nanjing, China) and randomly assigned to experimental groups. All animals were housed under a constant temperature and humidity, and maintained on a 12‐h light‐dark cycle with food and water available ad libitum. Each animal underwent intervention at 7–9 weeks of age. All mice were over 12 weeks old at the time of data collection. All animal experiments were approved by the Institutional Animal Care and Use Committee (IACUC) of the Southeast University (approval ID 20 220 303 005) and performed in accordance with the Animal Research: Reporting of In Vivo Experiments (ARRIVE) guidelines.

### Distal Middle Cerebral Artery Occlusion (dMCAO)

Focal cerebral ischemia was induced by direct occlusion of the distal middle cerebral artery.^[^ ^34^ ^]^ Briefly, anesthesia was induced with 3% isoflurane in 30% oxygen and 70% nitrous oxide via an anesthetic chamber and maintained with 1.5% isoflurane via a face mask using small animal anesthesia machines (RWD, R500). The right lateral aspect of the skull was exposed by cutting the skin between the right eye and ear, and separating the temporal muscle under a dissection microscope (OLYMPUS, SZ61). A 2‐mm diameter hole was made using a microdrill (RWD, 78 001) over the point downstream of the middle cerebral artery (MCA) lenticulostriate branches. The meninx was removed, and the artery was coagulated with the electrocoagulation forceps. After restoring the temporal muscle and suturing the wound, the mice were housed in a nursing box at 37 ± 0.5 °C until recovered from anesthesia.

### Adeno‐Associated Virus Injections

All surgical procedures were conducted under anesthesia using isoflurane (induction at 3%, maintenance at 1%‐2% vol vol^−1^), and all animals were placed in a stereotactic frame (RWD, 71000‐M) provided with a heating pad. For virus injection, a borosilicate pulled pipettes was inserted though the dura and 300 nL of virus were injected into the right somatosensory cortex (AP: −1.50 mm, ML: −1.80 mm, DV: −0.30 mm) at a rate of 30 nL min^−1^. After injection, the needle remained in place for 10 min to allow complete diffusion of the virus. For in vivo two‐photon imaging of neuronal dendritic spines, 400 nL of mixed virus solution (AAV2/9‐CMV‐CRE‐WPRE‐hGH polyA, 5.34 × 10^12^ vg mL^−1^, diluted 1:100000 in PBS and AAV2/9‐EF1α‐DIO‐EYFP‐EYFP‐WPREs, 5.81 × 10^12^ vg mL^−1^) was injected into the cortex. The AAV2/5‐GfaABC_1_D‐GCaMP6f‐WPRE, AAV2/5‐GFaABC_1_D‐mCherry‐5′miR‐30a‐shRNA (Ddx1)‐3′miR‐30a‐WPRE, AAV2/5‐GFaABC_1_D‐mCherry‐5′miR‐30a‐shRNA (scramble)‐3′miR‐30a‐WPRE, AAV2/5‐GfaABC_1_D‐Mito‐GCaMP6f‐P2A‐Pamcherry‐WPRE, AAV2/9‐hSyn‐GCaMP6s‐WPRE, AAV2/9‐CMV‐CRE‐WPRE‐hGH polyA, and AAV2/9‐EF1α‐DIO‐EYFP‐EYFP‐WPREs were constructed and packaged by BrainVTA.

### Cranial Window Implantation

Two weeks after the virus injection, the craniotomy surgery was performed on the mice. The anesthetized mice were fixed and a circular cranial window (4.8 mm in diameter) was centered over the targeted cortex. To remove bone pieces, the cranial window surface was cleaned with warm saline. A sterile circular glass coverslip (4.5–5.0 mm in diameter, 0.1 mm in thickness) was implanted into the craniotomy site carefully, and sealed with Tissue adhesive (No1469SB, 3 m). Then the coverslip and headplate were fixed over the cranial window with dental cement. The mice were allowed to recover for at least two weeks and were habituated with head‐fixation.

### Two‐Photon Imaging in Awake Animals

After two weeks of fixing the headplate, the mouse was restrained with a body holder, then the benchtop two‐photon microscope was used to find the region of viral infection. Two‐photon imaging was performed using the mTPM with a femtosecond fiber laser (≈35 mW at the objective, TVS‐FL‐01, Transcend Vivoscope Biotech Co., Ltd).^[^ ^32^ ^]^ Once the field of view was found with mTPM, the holder was glued to the headplate permanently and the objective was connected to the holder with three screws. The gap between the objective and the coverslip was filled with 1.5% low‐melting‐point agarose. The GINKGO‐MTPM software (GINKGO‐MTPM 1.0.28, Transcend Vivoscope Biotech Co., Ltd) was used for microscope control and image acquisition. All images were acquired at a frame of 10 Hz (512 × 512 pixel) and field of view was 420 µm × 420 µm. After imaging, the objective was dismounted by unscrewing and unplugging from the holder.

To ensure accurate identification of the peri‐infarct region for imaging, the following methodology was adopted: Initially, based on the experience, six fields of view (420 × 420 µm) were selected near the viral injection site for two‐photon imaging, which were considered as potential peri‐infarct regions. Subsequently, real‐time laser speckle contrast imaging (LSCI) was utilized to record baseline cortical blood flow in mice prior to dMCAO surgery and post‐surgery. Blood flow reductions in these six fields of view were compared. Through statistical analysis of the reduction of blood flow before and after the surgery, regions exhibiting over 35% reduction in blood flow were identified as the ischemic core.^[^ ^53^ ^]^ From the previously selected six fields of view, the areas adjacent were chosen to the ischemic core that demonstrated a 15–25% reduction in blood flow as the target fields of view for further imaging. If none of the initially selected six fields of view met these criteria, the mouse was excluded from the study.

### Image Processing of Calcium Imaging Data

Calcium imaging movies were corrected for movement in each plane separately utilizing either rigid motion‐correction with the TurboReg plugin in ImageJ or no‐rigid motion correction with the NoRMCorre algorithm in MATLAB (MathWorks).^[^ ^54^ ^]^ The imaging sequence was adjusted to exclude border regions that were not captured during the entire recording period. The noise was reduced with an isotropic (σ = 2) Gaussian Blur filter in Image J. The denoised imaging movies were subjected to a median filtered (radius: 5 pixels) and background correction. Then, for further analysis, only pixels with a median intensity or a peak intensity greater than the threshold of 5 (a.u.) were taken into consideration.

### Image Analysis of Astrocytic Calcium

The processed images were further analyzed for astrocytic calcium activity. For region of interest (ROI)‐based analysis, the ROI was detected by GECIquant software^[^ ^12^ ^]^ and followed by manually correction in ImageJ. The software identified ROIs corresponding to the soma (≥30 µm^2^), and microdomain (between 0.5 and 10 µm^2^). The fluorescence intensity time series were extracted from the segmented ROIs in Image J, and converted to ΔF/F values (ΔF/F = (Ft−F0) /F0). The basal fluorescence (F0) was determined during 20 s periods with no fluctuations. The fluorescence values of all pixels within each ROI were averaged to generate a single time course F(t). After applying spatial smoothing to the ΔF/F through averaging over a 300 ms sliding window, the standard deviation (s.d.) of each ROI was computed, and the events were identified based on amplitudes greater than twofold s.d. above F0. The amplitude, frequency, and other parameters of the astrocytic calcium signals were measured by custom‐written MATLAB code.

### Image Analysis of Astrocytic Mitochondrial Calcium

The AAV expressing GCaMP specifically on astrocyte mitochondria was utilized to detect the activity of Ca^2+^ in mitochondria (AAV2/5‐GfaABC_1_D‐Mito‐GCaMP6f‐P2A‐Pamcherry‐WPRE). As described in a previous study,^[^ ^20^ ^]^ the CaSCaDe algorithm was employed to analyze mitochondrial Ca^2+^ dynamics in astrocytic microdomains. The image processing for astrocytic mitochondrial imaging was conducted. The astrocytes in the field of view were manually segmented and analyzed one by one using the CaSCaDe software. The parameters and models in CaSCaDe are suggested from Amit et al.

### Image Analysis of Neuronal and Dendritic Spine Calcium

The open‐source software Suite2p was employed to detected ROIs of neurons and dendritic spines, and the calcium signals were extracted respectively. The outputs of Suite2p were manually inspected and subjected to additional analysis using customized scripts as described in “Image analysis of astrocytic calcium signaling”.

### Image Analysis of Neuronal Dendritic Spine Number

The number of neuronal dendritic spines was quantified using Image J software. Briefly, the dendritic spines surrounding the neuronal dendrites were manually defined by a 2‐µm‐diameter ROI, and the number of spines on a 30‐µm length dendrite was counted.

### Behavior Capture

A near infrared LED (780 nm) and camera were used to capture simple behaviors such as walking, standing concurrently with two‐photon fluorescence imaging for all experimental trials at a frame rate of 25 Hz. The prior protocols were followed for each behavioral test.^[^ ^34^ ^]^ The objective was mounted to the holder before the behavioral tests, and the mice were left in a dim imaging studio for five min to acclimate. Two entirely independent investigators who were blind to the experimental groupings conducted the behavioral tests and data processing.

### The Grid‐Walking Test

For the grid‐walking test, a wire mesh grid sized 32 cm × 20 cm × 50 cm (length, breadth, and height) was required, with the top of the grid made up of 12 mm squares. Each mouse walked independently and freely on the grid for 5 min. A camera was hidden beneath the grid to capture stepping errors (foot faults). In summary, it was defined as a foot fault: 1) if a step was not able to provide support and the foot went through the grid hole, or 2) if the mouse was resting with the grid at the level of its wrist. The numbers of foot faults and non‐faults for each limb were counted. A ratio was calculated as follows: number of foot faults/(number of foot faults + number of non‐faults) × 100%.

### The Cylinder Test

The cylinder test was applied to detect how forelimbs were used for wall exploration/press in a cylinder. Mice were placed inside a plastic cylinder (15 cm tall with a diameter of 10 cm) and videotaped for 5 min. The exploration/press score was calculated as follows: (number of right hand‐number of left hand) / (number of right hand + number of left hand + number of both hands).

### Laser Speckle Contrast Imaging of Awake Mice

After cranial window implantation, a body holder was used to restrain the awake mouse. Erythromycin eye ointment was applied to prevent eye dryness. The mice were positioned under the laser speckle contrast imaging system (RWD, RFLSI III) and let 5 min to acclimatize. Relative CBF changes in the cranial window were measured using LSCI software (RWD, V01.00.05.18305). Resolution: 3.4 µm pixel^−1^, Frame rate: 2 Hz, Laser intensity: 110 mW.

### Mitochondria Isolation for Peri‐Infarct Area Tissue of dMCAO Mice

Mitochondria from the peri‐infarct area of dMCAO mice were isolated using the Mitochondria Isolation Kit (ab110168, Abcam, USA). The isolation process involves three main steps: cell disruption, centrifugation to remove large particles, and centrifugation to isolate mitochondria. First, suspend the washed and minced tissue in Isolation Buffer and homogenize it. Then, centrifuge the homogenate at 1000 g for 10 min at 4 °C and collect the supernatant. Next, centrifuge the supernatant at 12 000 g for 15 min at 4 °C to obtain a pellet. Resuspend the pellet in Isolation Buffer with protease inhibitor (PI) and centrifuge again at 12 000 g for 15 min. Repeat the washing step by resuspending the pellet in Isolation Buffer with PI, then centrifuge at 12 000 g for 15 min. Finally, resuspend the pellet in Isolation Buffer with PI, aliquot, and freeze at −80 °C. The mitochondria were stored at −80 °C until further use. Perform mitochondrial assays according to the intended application.

### Mitochondria Isolation for Primary Mouse Astrocytes

Mitochondria from primary mouse astrocytes were isolated using the Mitochondria Isolation Kit (ab110170, Abcam, USA). The isolation procedure involves three main steps: cell rupture, centrifugation to remove large particles, and centrifugation to isolate mitochondria. First, freeze and thaw the cells to weaken the membranes, then suspend them in Reagent A at a concentration of 5.0 mg mL^−1^ and incubate on ice for 10 min. Next, homogenize the cells using a Dounce Homogenizer. Centrifuge the homogenate at 1000 g for 10 min at 4 °C, and collect and save the supernatant. Repeat the centrifugation step and save the second supernatant. Afterward, centrifuge the combined supernatants at 12 000 g for 10 min at 4 °C to collect the pellet. Finally, resuspend the pellet in Reagent C supplemented with protease inhibitors (PI), aliquot the suspension, and freeze at −80 °C. Perform mitochondrial assays according to the intended application.

### Protein Mass Spectrometry

Tissue from the peri‐infarct area was collected for protein mass spectrometry. Samples were processed using the U3000 nano nanoscale liquid chromatography system (Thermo Fisher Scientific, USA) coupled to a Q‐Exactive mass spectrometer (Thermo Fisher Scientific, USA) via the nano electrospray source. The false discovery rate (FDR) was controlled at the protein and peptide level using a fusion‐decoy database search strategy with a threshold ≤1.0%. MS/MS data were collected and stored in raw files using X calibur software (version 2.2, Thermo Fisher Scientific).

### Primary Mouse Astrocyte Cultures

P1 to P2 postnatal C57BL/6J mice were used to obtain the cells. Mice brains were mechanically dissociated, then major blood vessels and membranes were removed with gauze. The brains were digested with trypsin‐EDTA (25 200 056, Gibco). After digestion, the cells were plated on cell culture flasks precoated with poly‐L‐lysine. The cells were then incubated in CO_2_ (5%) at 37 °C.

### Oxygen Glucose Deprivation (OGD) Treatment

Primary mouse astrocytes were cultured with deoxygenated DMEM without glucose and FBS (11966‐025, Gibco) in a sealed chamber (MIC‐101, Billups‐Rothenburg) loaded with mixed gas consisting of 5% CO_2_ and 95% N_2_ for 5 min at 25 L min^−1^. The chamber was placed in a water‐jacketed incubator (Forma, Thermo Fisher Scientific). Astrocytes were incubated at 37 °C for 6 h. Astrocytes in the control group were cultured with normal DMEM and 10% FBS for the same period.

### Western Blot (WB) Analysis

Proteins were extracted in RIPA lysis buffer (P0013B, Beyotime), separated on sodium dodecyl sulfate polyacrylamide gels (8% and 12%), and electrophoretically transferred onto polyvinylidene fluoride membranes according to the previously described methods.^[^ ^35^ ^]^ The membranes were blocked with 5% nonfat dry milk in Tris‐buffered saline with Tween‐20 and probed with antibodies against DDX1 (11357‐1‐AP, Proteintech), and β‐actin (81115‐1‐RR, Proteintech) overnight at 4 °C. Then, they were incubated with HRP‐conjugated Affinipure Goat Anti‐Rabbit IgG (H+L) secondary antibody (SA00001‐2, Proteintech). Signals were detected by chemiluminescence and imaged using a Tanon digital image scanner (Tanon, 5200). Individual protein bands were quantified by densitometry using Image J software.

### Real‐Time PCR

Real‐time PCR for circSCMH1 and DDX1 was performed according to the previous studies using an Applied Biosystems Real‐time PCR System.^[^ ^35^ ^]^ First, total RNA was extracted using TRIzol reagent (15 596 026, Invitrogen) and treated with gDNA wiper. Then, the RNA was reverse transcribed using a HiScript Q RT SuperMix for qPCR Kit (R123‐01, Vazyme) and quantified using SYBR Green Real‐time PCR Master Mix. All samples were run in duplicate. The results were standardized to the control values of β‐actin. The sequences of the primers are listed in Table S3 (Supporting Information).

### RNA‐Binding Protein Immunoprecipitation (RIP)

Cultured primary astrocytes transfected with circSCMH1 lentivirus were collected with 0.5 mL lysis buffer according to the manufacturer's instructions of Magna RIP RNA‐binding protein immunoprecipitation kit (17‐700, Millipore). The magnetic beads were washed with RIP washing buffer. Next, the antibody of DDX1 (11357‐1‐AP, Proteintech) were added to the beads in RIP washing buffer and incubated for 1 h at room temperature. Then, RIP lysates were incubated with the antibody‐bound beads in RIP immunoprecipitation buffer overnight at 4 °C. After centrifuging for 5 min and discarding the supernatant, the pellet was resuspended in the immunoprecipitation buffer containing proteinase K in tubes and placed them at 55 °C for 30 min with shaking to digest the protein. Purified RNAs were examined the expression of circSCMH1 via qPCR.

### Fluorescence In Situ Hybridization (FISH)

According to the previously described methods,^[^ ^35^ ^]^ mouse primary astrocytes cultured on coverslips were fixed with 4% PFA for 20 min and incubated in PBS overnight at 4 °C, followed by processing to detect circSCMH1 expression. Next, the cells were permeabilized with 0.25% Triton X‐100 in PBS for 15 min and pre‐hybridized in hybridization buffer for 1 h at 37 °C. Then, hybridization buffer containing a biotin‐labeled circSCMH1 probe (50 n_M_, Invitrogen) was heated to 65 °C for 5 min and dripped onto the coverslips, followed by hybridization at 37 °C overnight. On the following day, the coverslips were washed three times in 2 × SSC and twice in 0.2 × SSC at 42 °C and then blocked with 1% BSA and 3% normal goat serum in PBS for 1 h at room temperature. The coverslips were subsequently incubated with FITC‐streptavidin (434 311, 1:200, Life Technology) overnight at 4 °C. After blocking with 1% BSA/1% Triton X‐100 in PBS (w v^−1^ for BSA and v v^−1^ for Triton X‐100) for 1 h at room temperature and then incubated with a rabbit anti‐DDX1 antibody (11357‐1‐AP, 1:500, Proteintech) for 24 h. Coverslips were washed three times in PBS and incubated with Alexa Fluor 594 goat anti‐rabbit IgG (A‐11012, 1:250, Invitrogen). Then the coverslips were washed twice with PBS and incubated with Hoechst 33 342 (H1399, Invitrogen) for 10 min at room temperature to visualize nuclei. The sections were finally washed once with DEPC water and mounted with 30% glycerine. Immunofluorescence images were captured via microscopy (Olympus, DP73). The circSCMH1 probe sequence, biotinylated at the 5′ end, was 5′‐ aaaTTGGAGGTGTGTAGGACTTTGGTGCCAGGTGG‐3′.

### Examination of Mitochondria by Transmission Electron Microscopy (TEM)

Anesthetized mice were transcranial perfused with a fixative solution containing 4% formaldehyde and 2.5% glutaraldehyde in PBS. The peri‐infarct area was isolated for further processing, and fixed in 2.5% glutaraldehyde in PBS. For EPON embedding, the fixed tissue was washed with 0.1 _M_ sodium cacodylate buffer, incubated with 1% OsO_4_ in 0.1 _M_ cacodylate buffer for 2 h at 4 °C and washed three times with 0.1 _M_ cacodylate buffer. Tissues were then dehydrated using an ascending ethanol series with 15 min incubation at 4 °C in each EtOH solution. Then tissues were transferred to propylene oxide and incubated in EPON (Sigma‐Aldrich) overnight at 4 °C. Tissues were placed in fresh EPON at RT for 2 h, followed by embedding at 37 °C for 24 h, 42 °C for 24 h, and 60 °C for 48 h. Ultrathin sections of 80 nm were cut using an ultramicrotome (Leica Microsystems, UC7) with a diamond knife and stained with 1.5% uranyl acetate at 37 °C for 15 min and lead citrate solution for 4 min. Electron micrographs were taken using a H‐7650 transmission electron microscopy (Hitachi) equipped with a 4 K 16‐bit camera (Gatan) and Digital Micrograph software (Gatan 830).

### TTC Staining and Measurement of Cerebral Infarction

Infarct volume was evaluated at 24 h after dMCAO according to the previously described methods.^[^ ^36^ ^]^ Briefly, the mice were anesthetized with isoflurane, and perfused with 0.01 _M_ PBS. After freezing at −20 °C for 6 min, each brain was coronally sectioned into five 1 mm slices with a brain matrix. The brain slices were incubated in 2% TTC (Sigma–Aldrich, T8877) at 37 °C for 10 min and subsequently fixed in 4% paraformaldehyde (PFA) to assess the size and extent of the infarction. The images were then analyzed using ImageJ software. To correct for brain swelling, the infarct area was determined by subtracting the non‐infarcted tissue area in the ipsilateral hemisphere from that of the intact contralateral hemisphere. Infarct volume was calculated by integrating the infarct areas across all slices of each brain.

### Immunostaining and Image Analysis

The brain tissues were sectioned into 30‐µm coronal slices, followed by incubation with 0.3% Triton X‐100 in PBS for 15 min and subsequent blocking with 10% normal goat serum (ZLI‐9056, ZSGB‐BIO) in 0.3% Triton X‐100 for 1 h at room temperature. The sections were incubated with a rabbit anti‐NeuN antibody (Abcam, ab177487) overnight. Sections were then incubated with Alexa Fluor 488 goat anti‐rabbit IgG (H + L) (Invitrogen, A‐11008) for 1 h at room temperature. The sections were mounted onto glass slides after being washed with PBS. The images were acquired using microscopy (OLYMPUS, DP73) and analyzed utilizing Image J software.

### Transfection and Preparation of EVs

The obtaining of EV was described in the previous study.^[^ ^34^ ^]^Briefly, a basic sequence was synthesized, which contained the 3′ half‐intron‐enox‐II fragment (splice acceptor or SA) of bacteriophage T4 td gene, a small space sequence, and an exon‐I splice donor 5′ half‐intron segment. The basic sequence, driven by a CMV promoter, was cloned into the multiple cloning sites of pEGFP‐N1 to generate circSCMH1 by replacing the small space sequence. The Vector plasmid was inserted without any sequence. Then, HEK293T (Chinese Academy of Sciences, Shanghai, China) cells were seeded in 225 cm^2^ flasks (431 082, Corning) and cultivated in 5% CO_2_. When reached ≈70% confluence, the cells were co‐transfected with the vector or circSCMH1 plasmid and RVGlamp2b (71 294, ADDGENE) using Lipofectamine 2000 (11 668 019, Invitrogen). After 48 h, hygromycin B (10 843 555 001, Roche) and puromycin (AK058, GPC Biotechnology) were added to the cell culture medium to obtain stable strains. HEK293T cells were grown in 5% CO_2_ in Dulbecco's modified Eagle's medium (DMEM, 10‐013‐CVR, Corning) supplemented with dialyzed fetal bovine serum (10% v v^−1^, 26 400 044, Gibco) with hygromycin B (200 ng mL^−1^, 108 435 550, Roche) and puromycin (100 µg mL^−1^, AK058, GPC Biotechnology). EVs were isolated from cell culture supernatants. Conditional medium was centrifuged for 5 min at 300 g to remove cells. Another centrifugation step was performed for 60 min at 10 000 g to remove shedding vesicles followed by 30 min at 3000 g to remove cell debris. The supernatants were filtered with 0.22 µm pore filters and centrifuged for 90 min at 200 000 g. The pellet was resuspended with PBS followed by another centrifugation step at 200 000 g to purify EVs.

### Statistics

All data for continuous are represented as means ± SEM. Statistical analysis was performed using GraphPad Prism 9.3.1 Software and the results were considered to be statistically significant if *P *< 0.05. Significance was assessed using Student's *t* test (2‐tail) for comparisons of two groups or Mann‐Whithey *U* test for nonnormally distributed data. Multigroup (three or more) comparisons were compared using one/two‐way ANOVA followed by the Holm‐Sidak post hoc test. Behavioral data collected at multiple, sequential time points (i.e., for a single animal at six‐time points) were analyzed using two‐way repeated‐measures ANOVA, followed by the Holm–Sidak post hoc test. Correlations were determined by calculating Pearson's correlation coefficients. The statistical analysis, *P* values, and samples sizes for the different experiments are described in the figure legends respective.

## Conflict of Interest

The authors declare no conflict of interest.

## Author Contributions

Z.‐Q.Z. and Y.B. contributed equally to this work. H.‐H.Y. conceived and supervised this project. H.‐H.Y., B.H., and Z.‐Q.Z. wrote the manuscript. Z.‐Q.Z. and W.X. established the dMCAO mice model. Z.‐Q.Z. and Y.B. offered important experimental methods. Y.B., H.R., Y.W., L.B., and X.L. performed molecular experiments. Z.‐Q.Z. analyzed data. Z.‐Q.Z., L.S., B.H., and Y.B. checked the data. X.‐Y.X., X.‐C.G., W.‐H.H., and Z.‐J.L. contributed to the experiment design and the discussion of the manuscript.

## Supporting information



Supporting Information

## Data Availability

The data that support the findings of this study are available from the corresponding author upon reasonable request.
