# Supplementary material for: Membrane Associated RNA‐Containing Vesicles Regulate Cortical Astrocytic Microdomain Calcium Transients in Awake Ischemic Stroke Mice
Source: Adv Sci (Weinh). 2024 Oct 23;11(46):2404391. doi: 10.1002/advs.202404391 (PMC11633488; doi:10.1002/advs.202404391)
Supplement: Supplementary file 1 — Supporting Information [file ADVS-11-2404391-s001.docx]

Supporting Information

**Membrane Associated RNA-Containing Vesicles Regulate Cortical Astrocytic Microdomain Calcium Transients in Awake Ischemic Stroke Mice**

*Zhongqiu Zhou, Ying Bai, Xiaochun Gu, Hui Ren, Wen Xi, Yu Wang, Liang Bian, Xue Liu,* *Ling Shen, Xianyuan Xiang, Wenhui Huang, Zhuojuan Luo, Bing Han^*^, Honghong Yao^*^*

**This Word file includes:**

Figure S1 to S11

Tables S1 to S3

**Supplementary figures and figure legends**

**
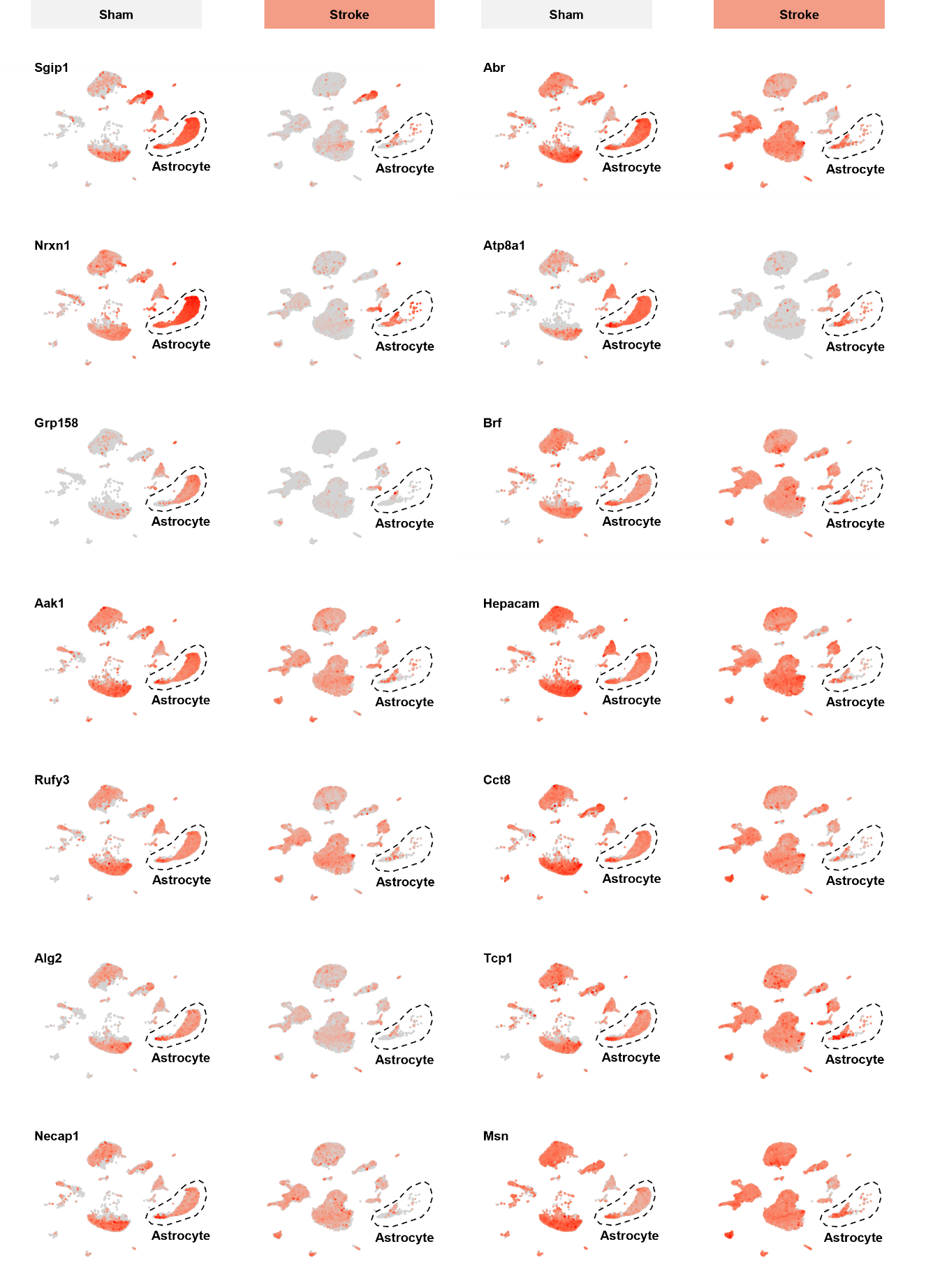
**

**Figure S1. Expression of astrocytic microdomain specific genes.**

Feature plots showing the expression of astrocytic microdomain specific genes in scRNA sequencing data. Black dashed outline represents astrocyte group.

**
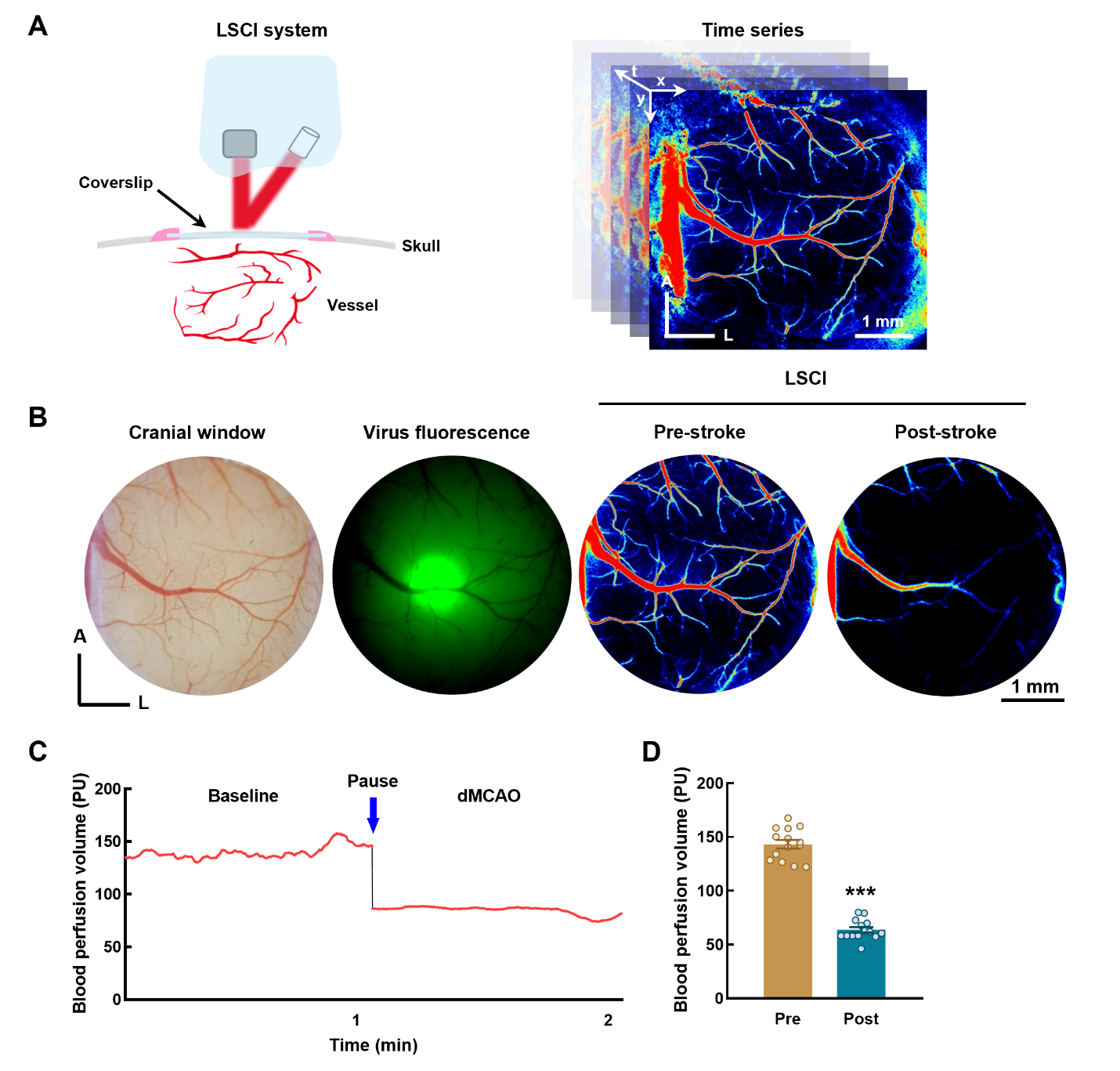
**

**Figure S2. Laser speckle contrast imaging for stroke mice.**

**A,** Left: Schematic of the approach for imaging of blood flow signals by cranial window in wake mice. Right: Representative time series images showing blood flow signals. **B,** Representative images from Pre-stroke and Post-stroke mice showing vasculature and blood flow signals. Left: Blood vessels in cranial window under light microscope. Middle: Blood vessels and fluorescence signals in cranial window under fluorescence microscope. Right: Blood flow signals in cranial window under LSCI. Scale bar, 1 mm. **C,** Recording area at the fluorescent region. **D,** Quantification of blood perfusion volume at the fluorescent region from Pre-stroke and Post-stroke mice. *n* = 14 mice. Data are shown as mean ± SEM. ^***^*P*<0.001 versus the Pre group using the Mann-Whitney *U* test. LSCI: Laser speckle contrast imaging; Pre: pre-stroke; Post: post-stroke.


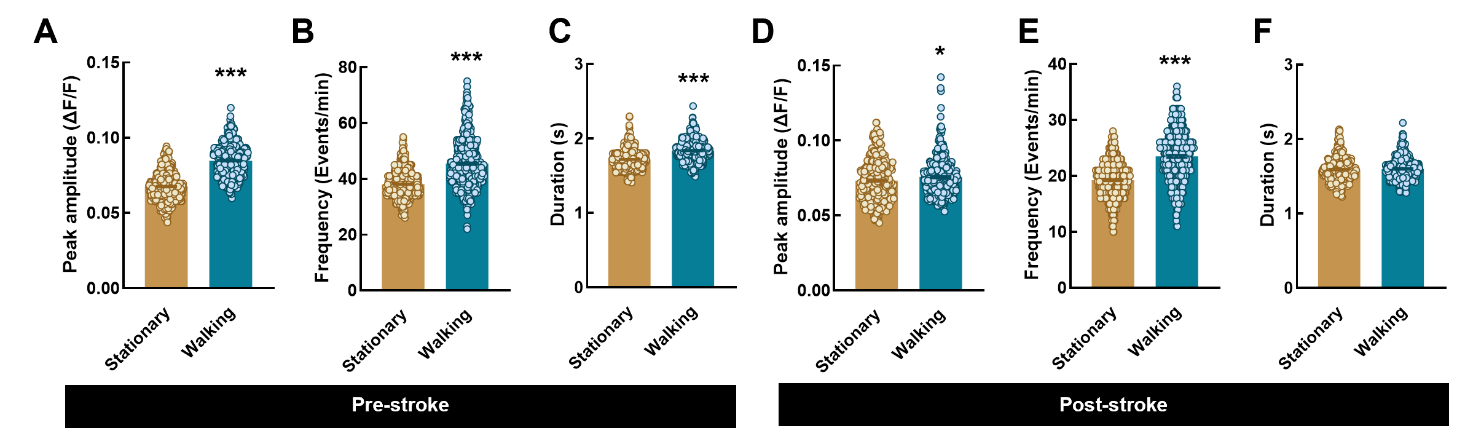


**Figure S3. Microdomain** **Ca^2+^ activity characteristics in stationary and walking mice before and after stroke.**

**A-C,** Quantification of pre-stroke microdomains GCaMP6f signal peak amplitude (**A**), mean frequency (**B**), and duration (**C**) derived from stationary and walking mice. Data are shown as mean ± SEM. ^***^*P*<0.001 versus stationary mice using the Mann-Whitney *U* test. **D-F,** Quantification of post-stroke microdomains GCaMP6f signal peak amplitude (**D**), frequency (**E**), and duration (**F**) derived from stationary and walking mice. Data are shown as mean ± SEM. ^*^*P*<0.05, ^***^*P*<0.001 versus stationary mice using the Mann-Whitney *U* test.


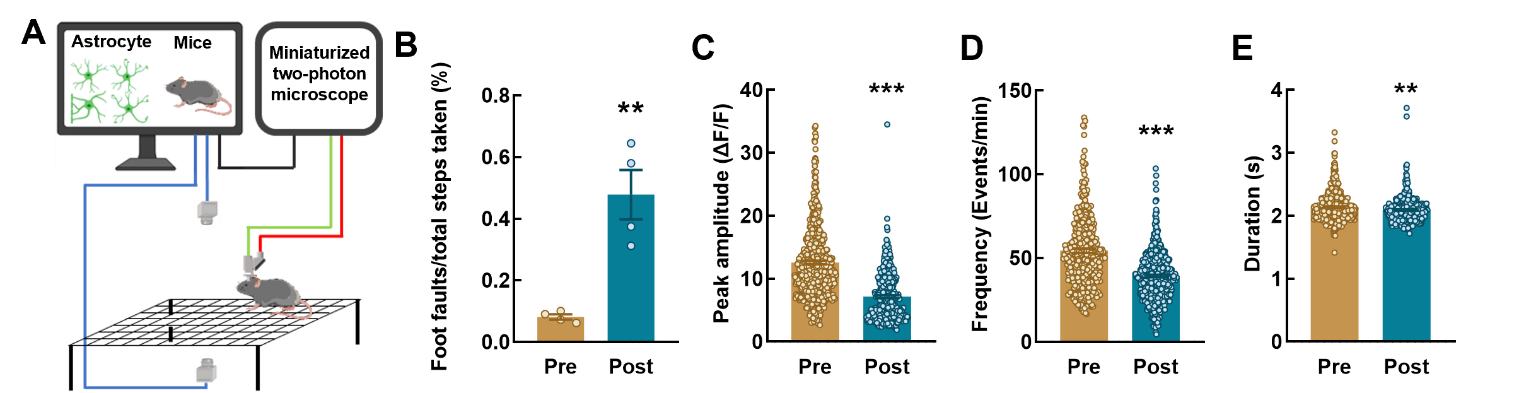


**Figure S4.** **Astrocytic microdomain Ca^2+^ transients are attenuated during grid-walking test after stroke.**

**A,** Schematic of the approach for imaging of astrocyte calcium signals in grid-walking test with mTPM. **B,** Assessment of forelimb motor function. Data are shown as mean ± SEM. ^**^*P*<0.01 versus the Pre group using the Mann-Whitney *U* test. **C-E,** Quantification of microdomains GCaMP6f signal peak amplitude (**C**), frequency (**D**) and duration (**E**) derived from 577 microdomains from Pre-stroke mice and 378 microdomains from post-stroke mice. Data are shown as mean ± SEM. ^**^*P*<0.01, ^***^*P*<0.001 versus the Pre group using the Mann-Whitney *U* test. Pre: pre-stroke; Post: post-stroke.

**
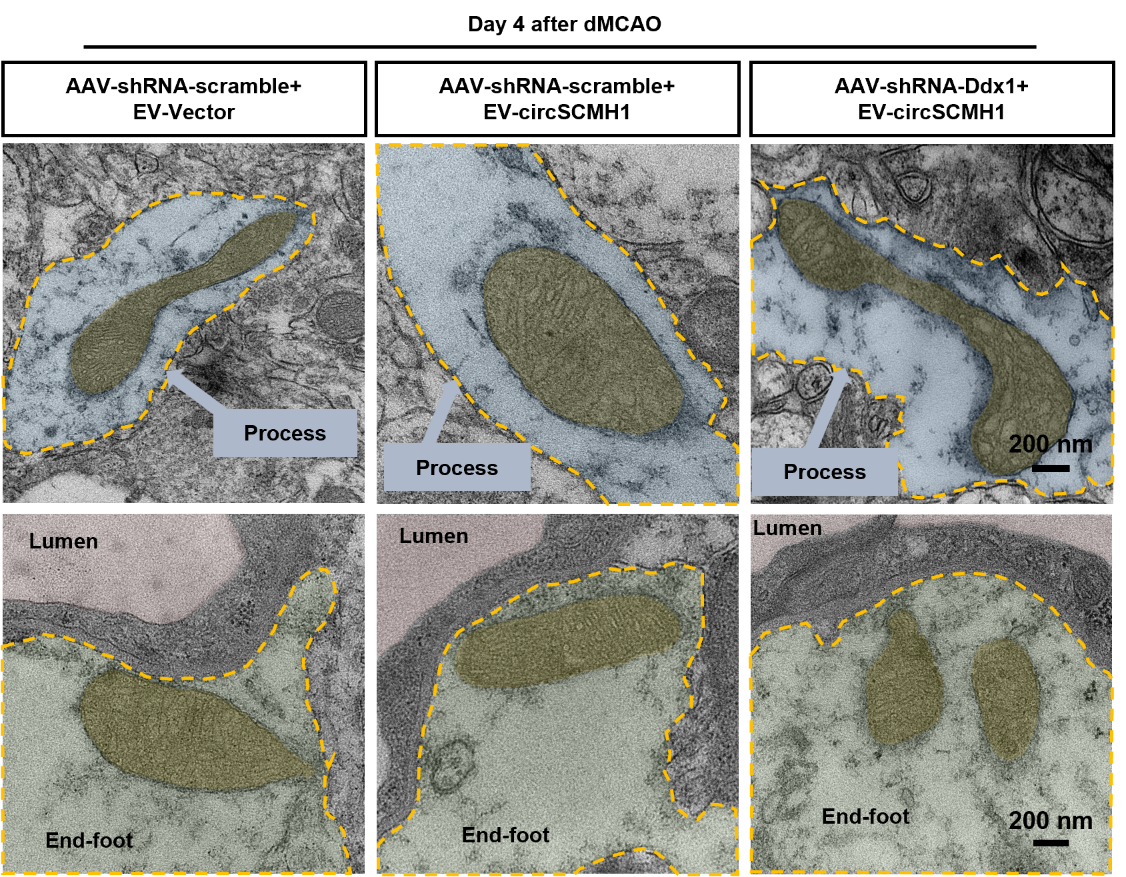
**

**Figure S5.** **The microdomain mitochondrial morphology by electron microscopy.**

TEM pictures of astrocytic end-feet and processes at 4 days after dMCAO, showing the morphology of perivascular mitochondria. Organelles are highlighted in different colors. Scale bars, 200 nm.

**
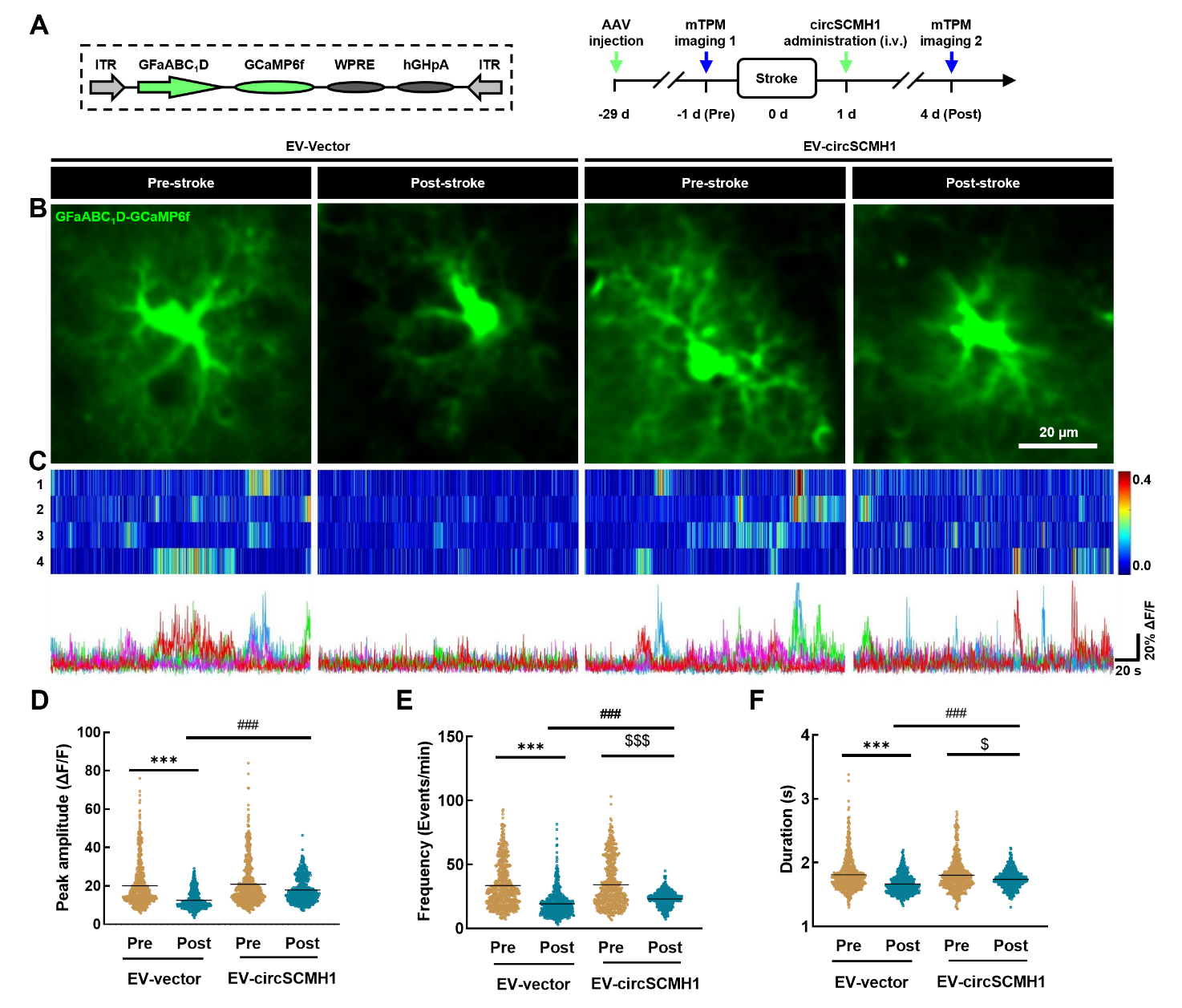
Figure S6.** **The administration of EV-circSCMH1 ameliorates the reduction in astrocytic microdomain Ca^2+^ transients induced by stroke.**

**A,** Schematic of EV-circSCMH1 administration and the approach for imaging of astrocyte calcium signals in vivo. **B,** Representative images from Pre-stroke and Post-stroke mice showing spontaneous GCaMP6f activity in astrocytes. Scale bar, 20 μm. **C,** Representative microdomain traces and heat map of GCaMP6f signal. Scale bars, 20 s (horizontal) and 20% ΔF/F (vertical). **D-F,** Quantification of microdomains GCaMP6f signal peak amplitude (**D**), frequency (**E**) and duration (**F**) in Pre + EV-Vector (656 microdomains, 3 mice), Post + EV-Vector (595 microdomains, 3 mice), Pre +EV-circSCMH1 (585 microdomains, 3 mice) and Post + EV-circSCMH1 (585 microdomains, 3 mice) group. Data are shown as mean ± SEM. ^***^*P*<0.001 versus the Pre + EV-vector group; ^###^*P*<0.001 versus the Post + EV-vector group; ^$^*P*<0.05, ^$$$^*P*<0.001 versus the Pre + EV-circSCMH1 group using 2-way ANOVA followed by Holm-Sidak post hoc multiple comparisons test. mTPM: mini two-photon microscope; Pre: pre-stroke; Post: post-stroke.

**
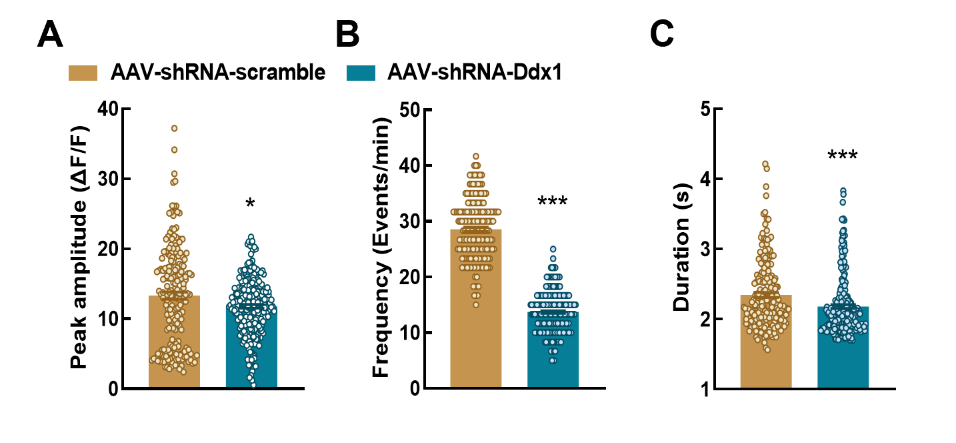
Figure S7.** **The specific knockdown of Ddx1 in astrocytes markedly attenuated microdomain Ca^2+^ transients.**

**A-C,** Quantification of microdomains GCaMP6f signal peak amplitude (**A**), frequency (**B**), and duration (**C**) in AAV-shRNA-scramble (193 microdomains, 3 mice) and AAV-shRNA-Ddx1 (238 microdomains, 3 mice) group. Data are shown as mean ± SEM. ^*^*P*<0.05, ^***^*P*<0.001 versus the AAV-shRNA-scramble group using the Student’s *t* test.

**
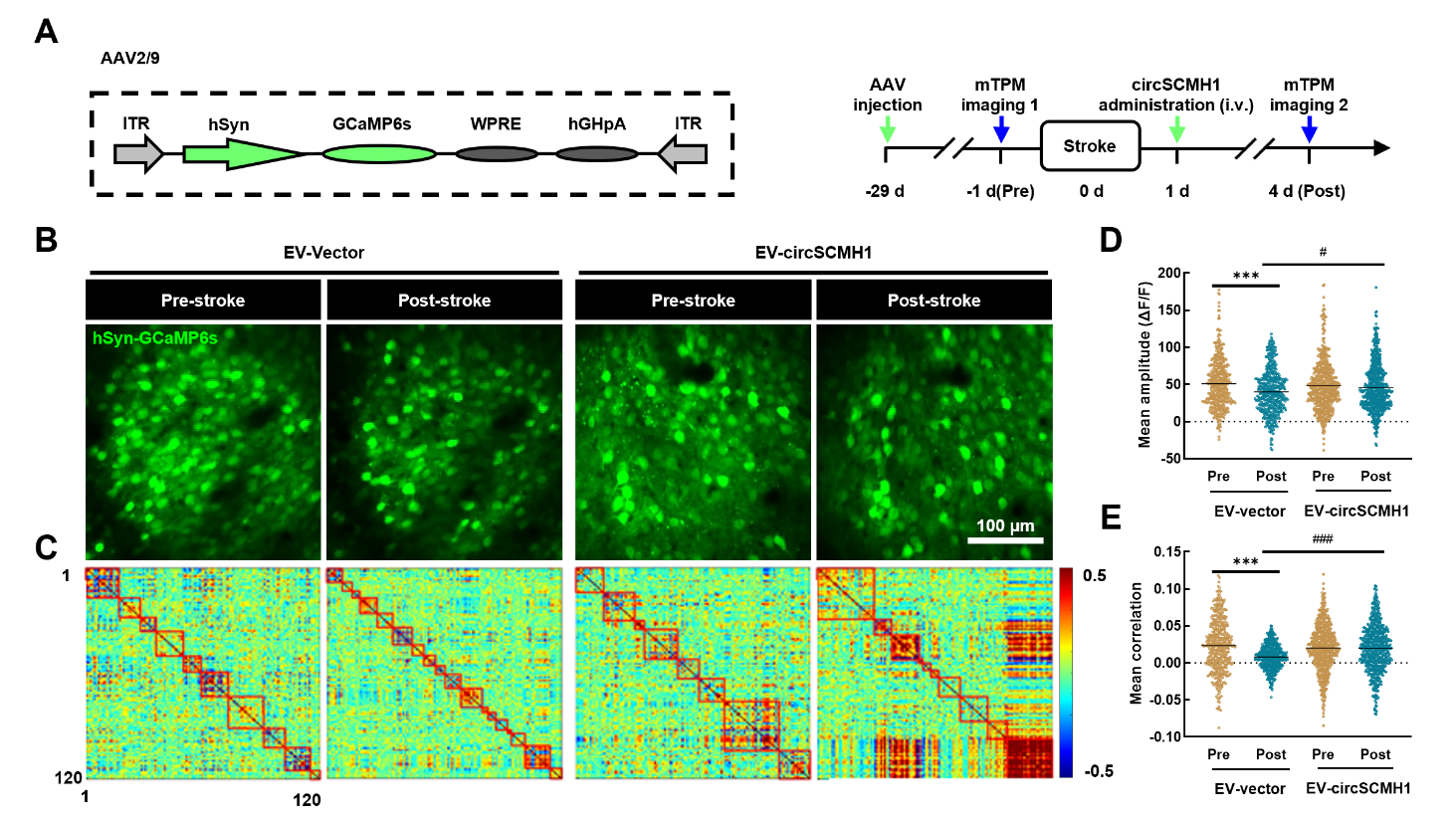
Figure S8.** **The administration of EV-circSCMH1 ameliorates the reduction in neuronal Ca^2+^ transients induced by stroke.**

**A,** Schematic of EV-circSCMH1 administration and the approach for imaging of neuron calcium signals *in vivo*. **B,** Representative images from Pre-stroke and Post-stroke mice showing spontaneous GCaMP6s activity in neurons. Scale bar, 20 μm. **C,** Map of correlation coefficient matrix among 120 neurons. **D-E,** Quantification of microdomains GCaMP6s signal mean amplitude (**D**) and mean correlation (**E**) in Pre + EV-Vector (447 neurons, 4 mice), Post + EV-Vector (447 neurons, 4 mice), Pre + EV-circSCMH1 (603 neurons, 4 mice), and Post + EV-circSCMH1 (603 neurons, 4 mice) group. Data are shown as mean ± SEM. ^***^*P*<0.001 versus the Pre + EV-vector group; ^#^*P*<0.05, ^###^*P*<0.001 versus the Post + EV-vector group using 2-way ANOVA followed by Holm-Sidak post hoc multiple comparisons test. mTPM: mini two-photon microscope; Pre: pre-stroke; Post: post-stroke.

**
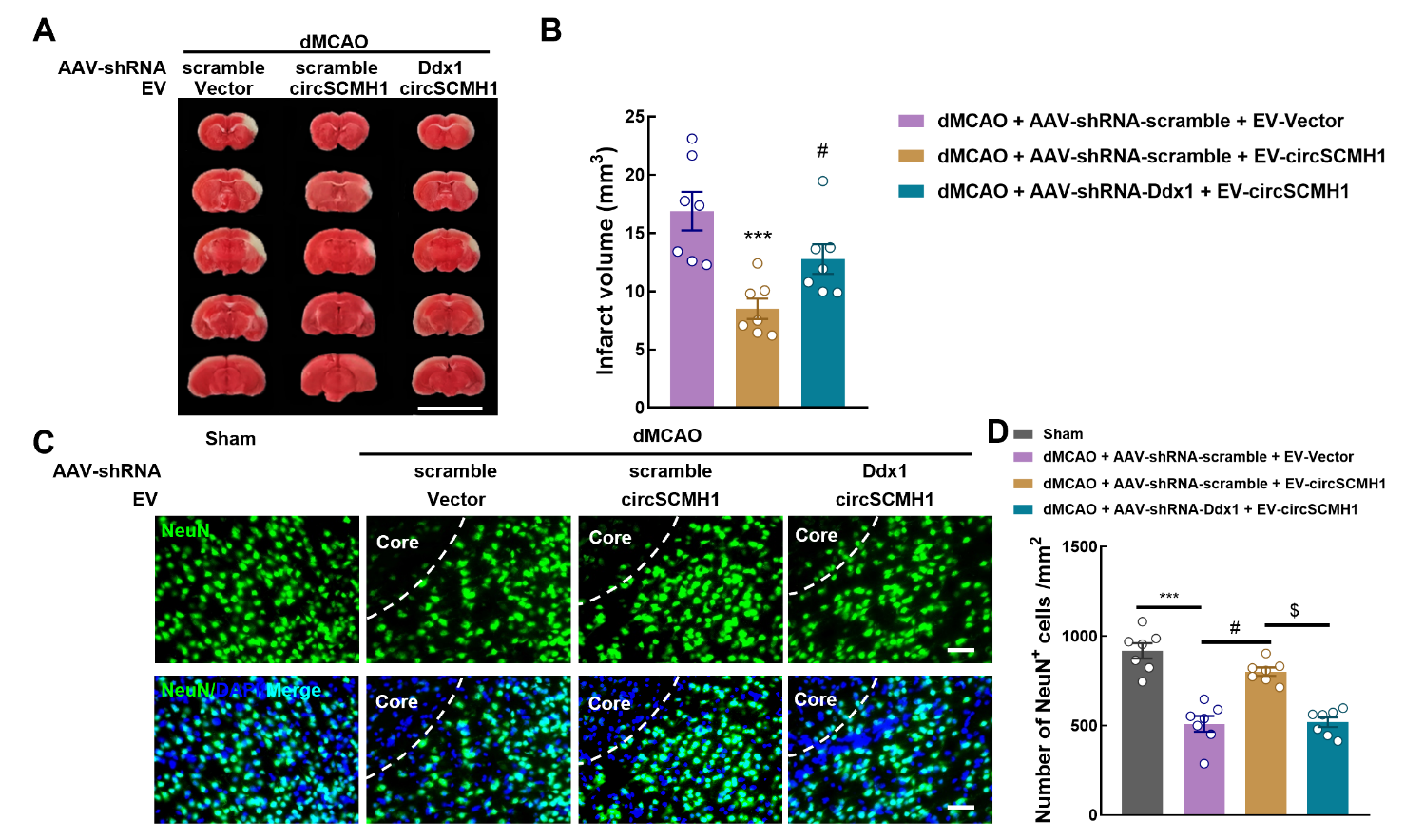
**

**Figure S9.** **The specific impact of these intracellular Ca²⁺ transients on the recovery of neuronal function.**

**A,** Representative of TTC-stained brain slices at day 4 after dMCAO. **B,** Bar graph showing infarct volume, *n*=7 animals per group. ^***^*P*<0.001 versus the dMCAO + AAV-shRNA-scramble + EV-vector group; ^#^*P*<0.05 versus the dMCAO + AAV-shRNA-scramble + EV-circSCMH1 group, using one-way ANOVA followed by the Holm-Sidak test. **C,** Representative NeuN^+^ cells in the peri-infarct cortex. Scale bar: 40 μm. **D,** Quantitation of NeuN^+^ cells in the brain. *n*=7 animals/group. ^***^*P*<0.001 versus the sham group, ^#^*P*<0.05 versus the dMCAO + AAV-shRNA-scramble + EV-Vector group, and ^$^*P*<0.05 versus dMCAO + AAV-shRNA-scramble + EV-circSCMH1 group using one-way ANOVA followed by the Holm-Sidak test.

**
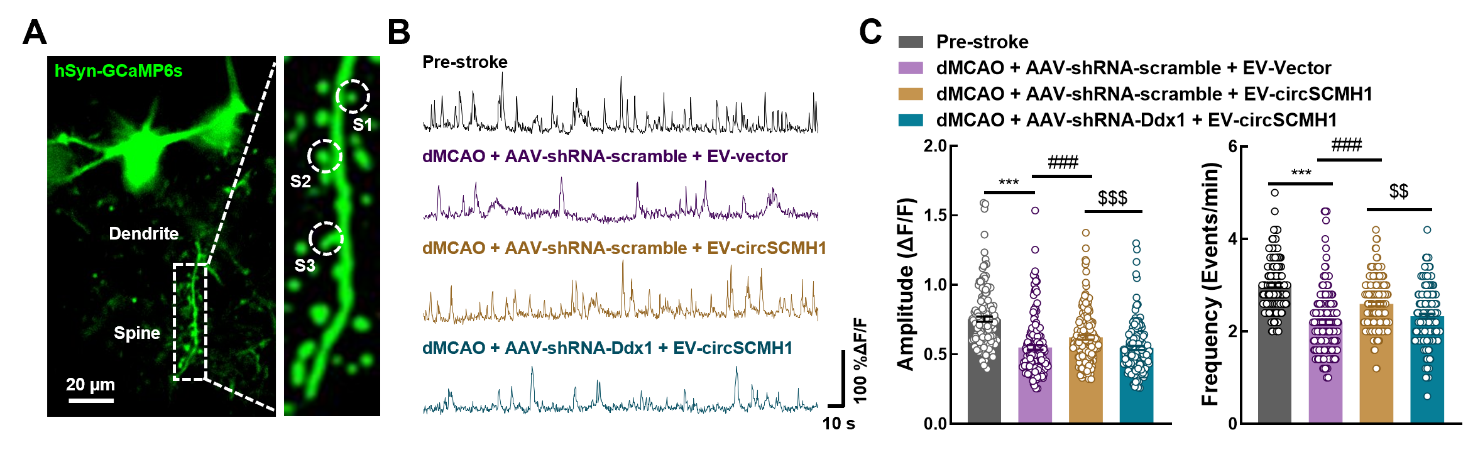
**

**Figure S10.** **The specific impact of these intracellular Ca²⁺ transients on neuronal synapses Ca²⁺ transients.**

**A,** Representative images of dendritic spine calcium signals. Scale bar, 20 μm. S: spine. **B,** Representative dendritic spine traces of ΔF/F GCaMP6f signal. Scale bars, 10 s (horizontal) and 100% ΔF/F (vertical). **C,** Quantification of dendritic spine GCaMP6s signals with amplitude and frequency. *n* =150 spines from 3 mice. ^***^*P*<0.001 versus the Pre-stroke group, ^###^*P*<0.001 versus the dMCAO + AAV-shRNA-scramble + EV-Vector group, and ^$$^*P*<0.01, ^$$$^*P*<0.001 versus dMCAO + AAV-shRNA-scramble + EV-circSCMH1 group using one-way ANOVA followed by the Holm-Sidak test.

**
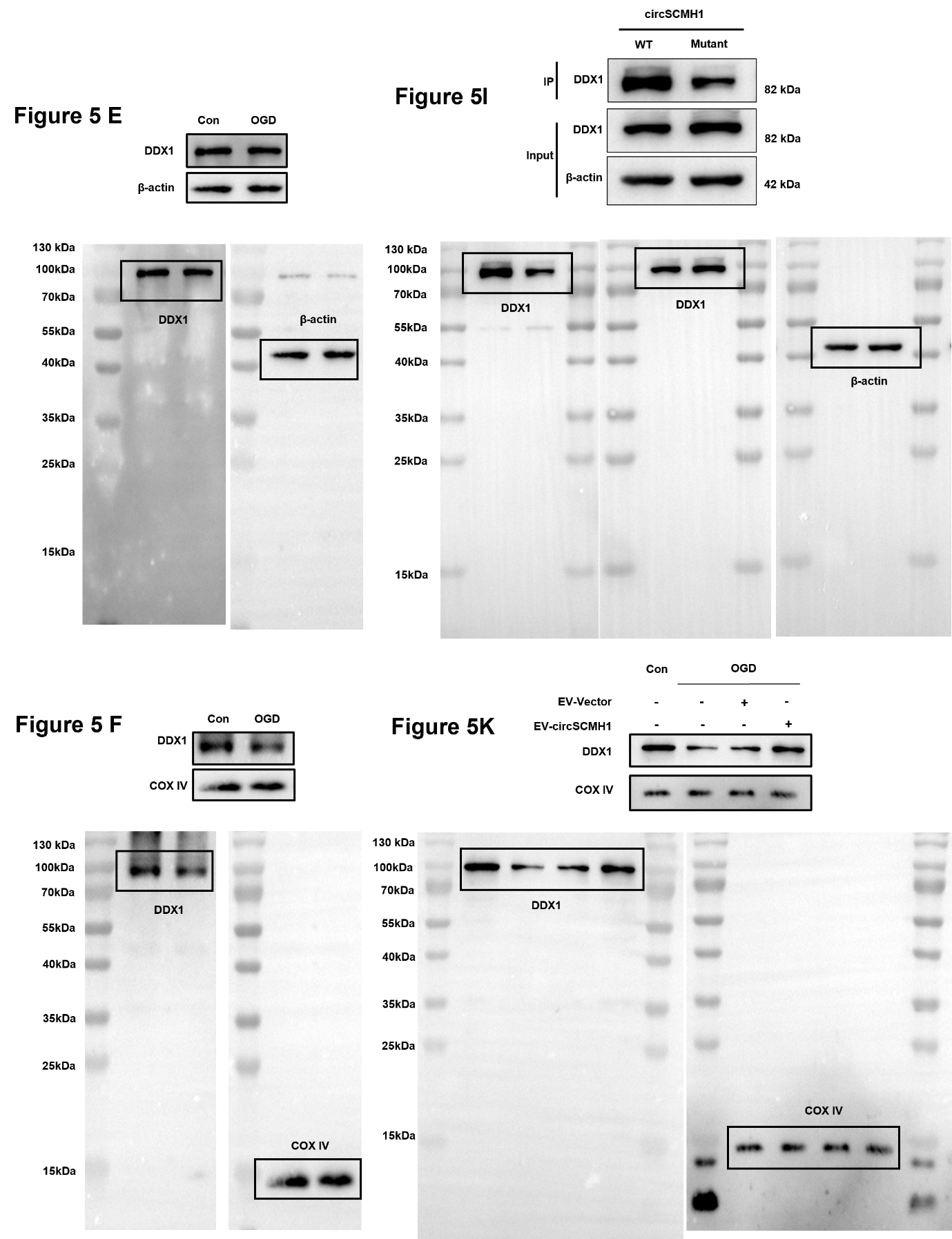
**

**Figure S11. Uncropped scans of western blot membranes used in Figure 5**

**Table S1. The significant changed proteins in mitochondrial proteomics analysis**

| **Accession** | **Gene Symbol** | **Sham** | **dMCAO** | **Fold change** | **P value** |
| --- | --- | --- | --- | --- | --- |
| NP_001039272.1 | Rbm15 | 144.333333 | 69.200000 | 2.085742 | 0.021541 |
| XP_006514367.1 | Anks1b | 136.200000 | 86.966667 | 1.566117 | 0.002140 |
| NP_001352047.1 | Tceal5 | 128.200000 | 83.633333 | 1.532882 | 0.000547 |
| NP_001028436.2 | Gls2 | 134.266667 | 89.500000 | 1.500186 | 0.001516 |
| XP_036009131.1 | - | 106.466667 | 71.600000 | 1.486965 | 0.023090 |
| XP_006538629.1 | Hp1bp3 | 112.300000 | 79.100000 | 1.419722 | 0.009098 |
| NP_034729.3 | Kcnab3 | 110.200000 | 79.433333 | 1.387327 | 0.038978 |
| NP_001186051.1 | Ghitm | 112.566667 | 81.400000 | 1.382883 | 0.011901 |
| NP_035930.1 | Cdh20 | 114.266667 | 82.666667 | 1.382258 | 0.016720 |
| XP_017168176.1 | Sorbs2 | 113.700000 | 83.366667 | 1.363854 | 0.002046 |
| NP_001156986.1 | Extl2 | 111.166667 | 81.533333 | 1.363451 | 0.009085 |
| NP_001347268.1 | Fam107a | 109.633333 | 80.733333 | 1.357969 | 0.033842 |
| NP_034002.2 | Cdk5r2 | 119.200000 | 88.566667 | 1.345879 | 0.025727 |
| NP_742120.2 | Crocc | 113.866667 | 85.800000 | 1.327117 | 0.000701 |
| NP_071312.1 | Nrgn | 117.300000 | 88.533333 | 1.324925 | 0.012701 |
| XP_011237794.1 | Ppp1r16b | 114.433333 | 86.666667 | 1.320385 | 0.003271 |
| XP_011240867.1 | AI593442 | 111.200000 | 84.433333 | 1.317015 | 0.011358 |
| NP_660254.1 | Lgi3 | 107.866667 | 82.033333 | 1.314913 | 0.001876 |
| NP_075661.1 | Ndufb9 | 120.800000 | 92.166667 | 1.310669 | 0.016057 |
| XP_006503647.2 | Pclo | 110.466667 | 84.433333 | 1.308330 | 0.011166 |
| NP_001300823.1 | Hmgb1 | 113.100000 | 86.866667 | 1.301995 | 0.009029 |
| XP_006529528.1 | Gigyf2 | 117.566667 | 91.133333 | 1.290051 | 0.027744 |
| NP_033904.2 | Bub3 | 117.200000 | 91.100000 | 1.286498 | 0.022350 |
| NP_001074266.1 | G3bp2 | 107.900000 | 84.966667 | 1.269910 | 0.002133 |
| NP_035482.1 | Sema7a | 110.466667 | 87.000000 | 1.269732 | 0.015395 |
| XP_006500681.1 | Lsm14b | 116.666667 | 91.933333 | 1.269036 | 0.041644 |
| NP_079870.1 | Zmat2 | 118.566667 | 93.600000 | 1.266738 | 0.030917 |
| NP_063928.2 | Abcb9 | 107.066667 | 84.566667 | 1.266062 | 0.004680 |
| NP_001075448.1 | Mecp2 | 114.566667 | 90.633333 | 1.264068 | 0.044522 |
| NP_113582.1 | Srrt | 107.066667 | 84.766667 | 1.263075 | 0.019316 |
| NP_808389.2 | Ranbp6 | 114.566667 | 91.266667 | 1.255296 | 0.022935 |
| XP_006509770.2 | Mast3 | 112.833333 | 89.900000 | 1.255098 | 0.030115 |
| NP_001136127.1 | Arpp19 | 111.900000 | 89.366667 | 1.252145 | 0.019247 |
| XP_030101204.1 | Ndufs7 | 108.566667 | 86.766667 | 1.251249 | 0.004729 |
| NP_001075124.1 | Dact3 | 107.000000 | 86.033333 | 1.243704 | 0.046548 |
| NP_776149.1 | Npr2 | 109.500000 | 88.500000 | 1.237288 | 0.038316 |
| NP_035040.1 | Nefl | 101.233333 | 81.966667 | 1.235055 | 0.008125 |
| NP_068685.1 | Tub | 107.500000 | 87.433333 | 1.229508 | 0.031167 |
| NP_932782.2 | Dagla | 108.633333 | 88.900000 | 1.221972 | 0.018831 |
| NP_067398.3 | Shank3 | 111.900000 | 91.600000 | 1.221616 | 0.017945 |
| NP_063922.2 | Rbm14 | 111.566667 | 91.433333 | 1.220197 | 0.023808 |
| NP_001380956.1 | - | 106.833333 | 87.566667 | 1.220023 | 0.039099 |
| XP_006508314.1 | Fam160a2 | 110.200000 | 90.333333 | 1.219926 | 0.013920 |
| XP_006525531.1 | Synpo | 106.266667 | 87.200000 | 1.218654 | 0.001588 |
| XP_030103582.1 | Kcnj3 | 108.600000 | 89.133333 | 1.218399 | 0.008781 |
| NP_034071.2 | Cox4i1 | 106.166667 | 87.266667 | 1.216578 | 0.005950 |
| NP_598596.2 | Ubac1 | 106.200000 | 87.366667 | 1.215567 | 0.022931 |
| XP_006527456.1 | Psd | 111.000000 | 91.333333 | 1.215328 | 0.020468 |
| NP_001290352.1 | Aldoc | 107.500000 | 88.466667 | 1.215147 | 0.019216 |
| NP_001028382.1 | Pls1 | 105.933333 | 87.200000 | 1.214832 | 0.004569 |
| XP_006510505.1 | Arhgap32 | 109.166667 | 89.966667 | 1.213412 | 0.017072 |
| NP_001153817.1 | Svip | 105.233333 | 86.833333 | 1.211900 | 0.047156 |
| NP_064370.2 | Habp4 | 111.300000 | 91.900000 | 1.211099 | 0.022791 |
| NP_031593.2 | Bsn | 111.600000 | 92.166667 | 1.210850 | 0.020540 |
| NP_001157012.1 | Plec | 104.733333 | 86.633333 | 1.208927 | 0.003248 |
| NP_033333.2 | Syt2 | 100.100000 | 82.933333 | 1.206994 | 0.019790 |
| XP_011237661.1 | Plcb4 | 106.033333 | 87.866667 | 1.206753 | 0.040785 |
| NP_291039.1 | Eif4h | 115.566667 | 95.800000 | 1.206333 | 0.027048 |
| XP_006499090.1 | Scn1a | 107.466667 | 89.100000 | 1.206135 | 0.037828 |
| NP_079965.2 | Ccdc51 | 112.600000 | 93.433333 | 1.205137 | 0.043496 |
| XP_011244270.1 | Kalrn | 113.066667 | 93.866667 | 1.204545 | 0.007650 |
| NP_659050.2 | Tmem143 | 106.066667 | 88.066667 | 1.204391 | 0.019763 |
| XP_030101237.1 | Anks1b | 113.933333 | 94.733333 | 1.202674 | 0.006724 |
| XP_006507783.1 | Atxn2l | 112.133333 | 93.300000 | 1.201858 | 0.025506 |
| NP_065585.1 | Mrps31 | 115.800000 | 96.433333 | 1.200830 | 0.033262 |
| NP_001386174.1 | - | 109.700000 | 91.400000 | 1.200219 | 0.007800 |
| NP_001158285.1 | Pet117 | 116.966667 | 97.533333 | 1.199248 | 0.017829 |
| XP_030098049.1 | Mag | 105.200000 | 87.800000 | 1.198178 | 0.039147 |
| NP_666212.3 | Ina | 99.933333 | 83.466667 | 1.197284 | 0.001990 |
| XP_030111196.1 | Eva1a | 109.300000 | 91.400000 | 1.195842 | 0.039520 |
| NP_080263.1 | Ndufa6 | 108.600000 | 90.866667 | 1.195158 | 0.012197 |
| NP_033931.4 | Car2 | 99.133333 | 82.966667 | 1.194857 | 0.015657 |
| XP_006515905.1 | Akap5 | 110.933333 | 92.900000 | 1.194116 | 0.006517 |
| XP_006499226.1 | Ube2e3 | 106.133333 | 89.033333 | 1.192063 | 0.013620 |
| NP_001342596.1 | Chchd3 | 110.700000 | 92.933333 | 1.191176 | 0.023633 |
| NP_001139437.1 | Dlgap2 | 110.033333 | 92.500000 | 1.189550 | 0.045074 |
| XP_017168102.1 | Cacna1e | 108.933333 | 91.633333 | 1.188796 | 0.023948 |
| XP_006532258.1 | Gabra1 | 107.200000 | 90.200000 | 1.188470 | 0.018543 |
| NP_780730.2 | Nyap1 | 113.900000 | 95.866667 | 1.188108 | 0.049587 |
| NP_077235.2 | Timmdc1 | 106.700000 | 89.866667 | 1.187315 | 0.042027 |
| NP_032717.2 | Nefm | 98.633333 | 83.133333 | 1.186447 | 0.020184 |
| XP_017169770.1 | Hdac5 | 112.300000 | 94.666667 | 1.186268 | 0.041382 |
| NP_080608.3 | Dnajc19 | 107.433333 | 90.566667 | 1.186235 | 0.040084 |
| NP_033969.2 | Cct6b | 112.566667 | 94.966667 | 1.185328 | 0.007542 |
| NP_001162130.1 | Ccdc127 | 108.533333 | 91.566667 | 1.185293 | 0.048340 |
| XP_006532073.2 | Aspa | 98.100000 | 82.766667 | 1.185260 | 0.031286 |
| NP_941020.1 | Dlgap3 | 110.200000 | 93.000000 | 1.184946 | 0.018299 |
| NP_001028361.1 | C77080 | 105.166667 | 88.766667 | 1.184754 | 0.027484 |
| XP_006530650.1 | Cacna1a | 105.800000 | 89.433333 | 1.183004 | 0.028834 |
| XP_006520332.1 | Adgrb1 | 107.666667 | 91.033333 | 1.182717 | 0.023068 |
| NP_997408.2 | Opa3 | 108.833333 | 92.033333 | 1.182543 | 0.035056 |
| XP_017172326.1 | Synj1 | 123.133333 | 104.233333 | 1.181324 | 0.022357 |
| NP_001029287.1 | Shank1 | 105.033333 | 89.033333 | 1.179708 | 0.030626 |
| NP_780421.2 | Tmem65 | 107.533333 | 91.466667 | 1.175656 | 0.025290 |
| XP_030110982.1 | Atp2b2 | 107.166667 | 91.500000 | 1.171220 | 0.017621 |
| XP_036009908.1 | - | 106.533333 | 91.100000 | 1.169411 | 0.008997 |
| NP_032313.2 | Rida | 106.066667 | 90.733333 | 1.168993 | 0.023649 |
| NP_058054.2 | Atp5b | 106.833333 | 91.400000 | 1.168855 | 0.018331 |
| NP_079627.3 | Chchd6 | 110.000000 | 94.333333 | 1.166078 | 0.031031 |
| NP_001369144.1 | Ndufa13 | 104.300000 | 89.500000 | 1.165363 | 0.000806 |
| NP_032446.2 | Kcnb1 | 104.833333 | 90.000000 | 1.164815 | 0.024282 |
| XP_030101071.1 | Atp5d | 112.133333 | 96.333333 | 1.164014 | 0.044028 |
| NP_080119.1 | Ndufb7 | 108.866667 | 93.600000 | 1.163105 | 0.002668 |
| XP_006515859.1 | Ppp1r13b | 118.766667 | 102.133333 | 1.162859 | 0.001532 |
| NP_080286.2 | Lipt2 | 104.566667 | 89.933333 | 1.162713 | 0.031834 |
| NP_080175.1 | Uqcrc2 | 106.400000 | 91.566667 | 1.161995 | 0.043665 |
| XP_006503645.2 | Pclo | 105.233333 | 90.600000 | 1.161516 | 0.013528 |
| YP_001686701.1 | COX2 | 106.766667 | 92.000000 | 1.160507 | 0.005774 |
| NP_031476.3 | Slc25a4 | 104.200000 | 89.833333 | 1.159926 | 0.034706 |
| NP_001342409.1 | Slc12a5 | 104.566667 | 90.200000 | 1.159276 | 0.000314 |
| NP_080194.1 | Ccdc43 | 109.466667 | 94.533333 | 1.157969 | 0.012093 |
| NP_001077356.1 | Pars2 | 107.833333 | 93.166667 | 1.157424 | 0.022067 |
| XP_006498923.1 | Mertk | 106.166667 | 91.733333 | 1.157340 | 0.000348 |
| NP_079683.2 | Uqcrc1 | 106.133333 | 91.800000 | 1.156137 | 0.047917 |
| NP_034072.2 | Cox5b | 107.266667 | 92.833333 | 1.155476 | 0.007226 |
| XP_036008753.1 | - | 101.966667 | 88.300000 | 1.154775 | 0.049926 |
| NP_077176.1 | Tomm20 | 109.466667 | 94.800000 | 1.154712 | 0.001431 |
| NP_062265.2 | Tyro3 | 107.133333 | 92.866667 | 1.153625 | 0.024644 |
| XP_006495755.1 | Erbb4 | 103.400000 | 89.733333 | 1.152303 | 0.011969 |
| XP_011249307.1 | Tenm1 | 110.733333 | 96.100000 | 1.152272 | 0.023397 |
| NP_079736.1 | Tmem126a | 106.000000 | 92.033333 | 1.151757 | 0.026571 |
| NP_062624.2 | Gosr2 | 98.033333 | 85.133333 | 1.151527 | 0.045423 |
| XP_030101491.1 | Mapt | 109.000000 | 94.666667 | 1.151408 | 0.004107 |
| NP_038823.2 | Atp5l | 108.066667 | 94.000000 | 1.149645 | 0.033632 |
| NP_081226.1 | Ociad2 | 109.900000 | 95.633333 | 1.149181 | 0.033586 |
| NP_001156417.1 | Mtx3 | 103.433333 | 90.033333 | 1.148834 | 0.006414 |
| XP_036013945.1 | - | 108.833333 | 94.833333 | 1.147627 | 0.042662 |
| XP_011241424.1 | Grik2 | 110.533333 | 96.333333 | 1.147405 | 0.015076 |
| NP_848794.1 | Zfp365 | 105.666667 | 92.133333 | 1.146889 | 0.046466 |
| NP_081186.1 | Tnik | 108.900000 | 95.100000 | 1.145110 | 0.027060 |
| NP_067277.2 | Stk32c | 105.966667 | 92.566667 | 1.144761 | 0.047544 |
| NP_031447.1 | Adssl1 | 102.600000 | 89.666667 | 1.144238 | 0.027587 |
| NP_001164257.1 | Mthfd1l | 110.166667 | 96.366667 | 1.143203 | 0.018555 |
| NP_001365996.1 | Slc25a23 | 106.666667 | 93.400000 | 1.142041 | 0.005449 |
| NP_058033.2 | Lxn | 105.433333 | 92.333333 | 1.141877 | 0.035364 |
| NP_789819.2 | Arglu1 | 101.500000 | 88.966667 | 1.140877 | 0.016130 |
| XP_006518557.1 | Camk2g | 107.833333 | 94.633333 | 1.139486 | 0.011101 |
| NP_079645.1 | Mrps36 | 101.733333 | 89.333333 | 1.138806 | 0.014967 |
| NP_001361626.1 | Slc44a1 | 106.066667 | 93.166667 | 1.138462 | 0.028831 |
| XP_006540500.1 | Sptbn4 | 104.066667 | 91.433333 | 1.138170 | 0.007821 |
| XP_030109871.1 | Psph | 101.066667 | 88.800000 | 1.138138 | 0.020508 |
| NP_001357604.1 | Dlg4 | 107.400000 | 94.400000 | 1.137712 | 0.032423 |
| NP_659061.2 | Tnrc6b | 115.400000 | 101.466667 | 1.137319 | 0.027448 |
| NP_080425.3 | Nudcd1 | 102.700000 | 90.366667 | 1.136481 | 0.018056 |
| NP_001180195.2 | Mdga2 | 104.966667 | 92.400000 | 1.136003 | 0.023598 |
| NP_035622.1 | Stk11 | 102.500000 | 90.266667 | 1.135524 | 0.029716 |
| NP_065630.1 | Jph3 | 111.933333 | 98.600000 | 1.135227 | 0.018497 |
| NP_082553.2 | Shisa9 | 109.000000 | 96.100000 | 1.134235 | 0.031096 |
| NP_001342186.1 | Gpr162 | 106.766667 | 94.133333 | 1.134207 | 0.026463 |
| NP_035018.1 | Ndufs6 | 107.733333 | 95.000000 | 1.134035 | 0.026323 |
| NP_766292.2 | Dnajc11 | 106.366667 | 93.800000 | 1.133973 | 0.009238 |
| NP_038703.3 | Sptb | 97.866667 | 86.333333 | 1.133591 | 0.005339 |
| XP_006503165.1 | Rims3 | 101.200000 | 89.400000 | 1.131991 | 0.026365 |
| NP_001278084.1 | Tomm34 | 105.633333 | 93.500000 | 1.129768 | 0.005949 |
| XP_006506926.1 | Plekha5 | 108.900000 | 96.400000 | 1.129668 | 0.022427 |
| XP_036014338.1 | - | 108.366667 | 95.966667 | 1.129212 | 0.001951 |
| NP_922922.1 | Wipf2 | 104.266667 | 92.600000 | 1.125990 | 0.012684 |
| XP_036011414.1 | - | 100.700000 | 89.433333 | 1.125978 | 0.047394 |
| XP_006539838.1 | Shisa7 | 105.533333 | 93.800000 | 1.125089 | 0.019302 |
| XP_030109960.1 | Cit | 105.866667 | 94.100000 | 1.125044 | 0.014157 |
| NP_079852.1 | Ptpmt1 | 104.266667 | 92.733333 | 1.124371 | 0.044601 |
| XP_036016008.1 | - | 104.800000 | 93.233333 | 1.124061 | 0.019057 |
| XP_006524376.1 | Caskin1 | 107.133333 | 95.400000 | 1.122991 | 0.015223 |
| NP_001346432.1 | Mtus2 | 103.733333 | 92.500000 | 1.121441 | 0.029323 |
| XP_017172004.1 | Scn8a | 102.066667 | 91.100000 | 1.120381 | 0.031142 |
| NP_001185933.1 | Golga5 | 105.433333 | 94.166667 | 1.119646 | 0.002095 |
| XP_030099428.1 | Acsf3 | 106.766667 | 95.366667 | 1.119539 | 0.014943 |
| NP_001025153.2 | Sec23ip | 105.166667 | 94.000000 | 1.118794 | 0.021440 |
| NP_001185564.1 | Map7 | 104.866667 | 93.733333 | 1.118777 | 0.030070 |
| NP_001355807.1 | Sytl2 | 104.733333 | 93.633333 | 1.118548 | 0.026933 |
| XP_011241850.1 | Atp2b1 | 104.733333 | 93.633333 | 1.118548 | 0.036396 |
| XP_006516371.1 | Ppp4r4 | 112.433333 | 100.566667 | 1.117998 | 0.023680 |
| XP_017170657.1 | Eml1 | 103.166667 | 92.400000 | 1.116522 | 0.010833 |
| XP_006532601.1 | Bcas3 | 104.600000 | 93.733333 | 1.115932 | 0.019359 |
| NP_032248.3 | Hccs | 109.433333 | 98.066667 | 1.115908 | 0.044687 |
| XP_036011805.1 | - | 103.633333 | 92.933333 | 1.115136 | 0.016268 |
| NP_848734.1 | Timm29 | 103.266667 | 92.633333 | 1.114789 | 0.018556 |
| NP_598801.1 | Ddx1 | 103.333333 | 92.700000 | 1.114707 | 0.021376 |
| NP_076027.1 | Agk | 103.233333 | 92.633333 | 1.114430 | 0.029729 |
| NP_080339.1 | Uqcc2 | 110.433333 | 99.100000 | 1.114363 | 0.020258 |
| NP_001240761.1 | Fibp | 107.166667 | 96.266667 | 1.113227 | 0.022870 |
| XP_006514364.1 | Anks1b | 108.866667 | 97.900000 | 1.112019 | 0.014333 |
| XP_036013237.1 | - | 106.900000 | 96.466667 | 1.108155 | 0.028194 |
| NP_061289.1 | Mpc1 | 104.566667 | 94.366667 | 1.108089 | 0.013530 |
| NP_001139302.1 | Plcb1 | 99.500000 | 89.800000 | 1.108018 | 0.037816 |
| NP_034490.1 | Gstm5 | 100.600000 | 90.833333 | 1.107523 | 0.045926 |
| XP_006498793.1 | Ext2 | 105.666667 | 95.466667 | 1.106844 | 0.032911 |
| XP_036017893.1 | - | 107.100000 | 96.766667 | 1.106786 | 0.014810 |
| NP_001342519.1 | Mfn2 | 102.433333 | 92.633333 | 1.105793 | 0.016040 |
| NP_062303.2 | Cacng3 | 105.966667 | 96.033333 | 1.103436 | 0.014878 |
| XP_006495827.1 | Myo1b | 103.933333 | 94.300000 | 1.102156 | 0.000416 |
| NP_082645.1 | Mon1a | 106.300000 | 96.633333 | 1.100034 | 0.041035 |
| NP_109632.2 | Fcrls | 66.266667 | 132.566667 | 0.499874 | 0.010729 |
| NP_033140.1 | S100a9 | 60.433333 | 121.566667 | 0.497121 | 0.016057 |
| NP_032720.2 | Ngp | 53.500000 | 107.700000 | 0.496750 | 0.042521 |
| NP_035837.1 | Vtn | 58.700000 | 119.666667 | 0.490529 | 0.005098 |
| NP_035831.2 | Vim | 63.766667 | 133.533333 | 0.477534 | 0.001235 |
| NP_001104518.1 | Fga | 59.633333 | 125.033333 | 0.476939 | 0.001863 |
| NP_033278.2 | Serpina3n | 57.533333 | 124.300000 | 0.462859 | 0.000764 |
| NP_032122.1 | Gc | 53.400000 | 119.066667 | 0.448488 | 0.000457 |
| NP_034572.1 | Hmox1 | 63.200000 | 142.533333 | 0.443405 | 0.014980 |
| NP_034407.2 | Gfap | 57.866667 | 131.166667 | 0.441169 | 0.000110 |
| NP_598738.1 | Trf | 57.400000 | 132.200000 | 0.434191 | 0.003683 |
| NP_059067.2 | Hpx | 53.300000 | 126.166667 | 0.422457 | 0.012585 |
| XP_006496689.1 | Serpinc1 | 54.966667 | 130.366667 | 0.421631 | 0.001574 |
| XP_006506523.1 | Tmem176b | 58.166667 | 139.200000 | 0.417864 | 0.006782 |
| NP_031496.2 | Apod | 50.333333 | 129.966667 | 0.387279 | 0.003885 |
| NP_001365804.1 | Lgmn | 56.166667 | 145.533333 | 0.385937 | 0.000033 |
| NP_059068.1 | Lyz2 | 49.766667 | 131.733333 | 0.377783 | 0.006275 |
| NP_001239498.1 | Serpina1a | 45.733333 | 134.733333 | 0.339436 | 0.002544 |
| NP_038503.4 | Apoh | 46.433333 | 137.600000 | 0.337452 | 0.000561 |
| NP_001191130.1 | Spp1 | 47.300000 | 144.733333 | 0.326808 | 0.000982 |
| XP_030106272.1 | Ttr | 46.633333 | 144.433333 | 0.322871 | 0.004151 |
| NP_034298.1 | F2 | 40.366667 | 126.033333 | 0.320286 | 0.005202 |
| NP_035588.2 | Serpina3k | 41.300000 | 138.933333 | 0.297265 | 0.001399 |
| NP_034835.1 | Lgals3 | 44.366667 | 149.500000 | 0.296767 | 0.002928 |
| NP_001155187.1 | Tgm1 | 44.033333 | 151.600000 | 0.290457 | 0.000158 |
| NP_619613.2 | Stab1 | 44.066667 | 155.966667 | 0.282539 | 0.005136 |
| XP_006509374.1 | Msr1 | 39.566667 | 142.466667 | 0.277726 | 0.001055 |
| NP_033784.2 | Alb | 34.466667 | 151.933333 | 0.226854 | 0.000374 |

**Table S2. The prediction of DDX1-binding circRNA**

| **circRNA** | **Interaction_Propensity** |
| --- | --- |
| circSCMH1 | 48.31 |
| circHECTD1 | 43.92 |
| circTLK1 | 48.02 |
| circDLGAP4 | 15.19 |

**Table S3. The primer sequences used for qPCR analysis**

| **List of oligonucleotide sequences** | **5' > 3'** |
| --- | --- |
| circSCMH1-F | CTACTGGTGCCGCTTTGACT |
| circSCMH1-R | GGCACCTGTCAATCCAACGA |
| DDX1 (mouse)-F | CTCCGAAATGGGTGTTATGCC |
| DDX1 (mouse)-R | GCCATGAGTACATCCCCTCCT |
| GAPDH (mouse)-F | AGGTCGGTGTGAACGGATTTG |
| GAPDH (mouse)-R | TGTAGACCATGTAGTTGAGGTCA |
| β-actin (mouse)-F | GGCTGTATTCCCCTCCATCG |
| β-actin (mouse)-R | CCAGTTGGTAACAATGCCATGT |
| Sgip1 (mouse)-F | ACGAGCCACCCTACCATAG |
| Sgip1 (mouse)-R | GTGCCCCATTGCTTTTCTTCG |
| Nrxn1 (mouse)-F | AACGGACTGATGCTTCACACA |
| Nrxn1 (mouse)-R | GATATTGTCACCTGACGCAGATT |
| Gpr158 (mouse)-F | TGCTCCAAGAAGGGCTTAGG |
| Gpr158 (mouse)-R | TCCCTGTATTCCCTGAGGACT |
| Aak1 (mouse)-F | TGTTGGCGGAAGGTGGATTC |
| Aak1 (mouse)-R | GACAAACATACGTTTCAAGGCAC |
| Rufy3 (mouse)-F | TAACAGCGAGTGTAAAAGACCTC |
| Rufy3 (mouse)-R | AGACCAGCGATAATAGCTCCTT |
| Alg2 (mouse)-F | ATGGCCGAAAACCTGTACCG |
| Alg2 (mouse)-R | CTCCGCCTATACCCATGTCTG |
| Necap1 (mouse)-F | CGTCAGCGTCTACCGGATTC |
| Necap1 (mouse)-R | AGTTTGATGTAGGCAATCTTCCC |
| Abr (mouse)-F | ATGCCGTACATCGACGAGTC |
| Abr (mouse)-R | AGGTGCCAATCCCTCAGGT |
| Atp8a1 (mouse)-F | TTAGACAAGGCTTACCGGCAA |
| Atp8a1 (mouse)-R | CTTTCACACTCGATTCTGCCA |
| Braf (mouse)-F | TGATGCGCTGTCTTCGGAAAT |
| Braf (mouse)-R | GCCAGGCTCAAAATCAAACACT |
| Hepacam (mouse)-F | AGCCTGCCTGTCAAGATCAC |
| Hepacam (mouse)-R | CCAAGGAGTTTTGCTTCTCCAAC |
| Cct8 (mouse)-F | ATTTCTCGGGATTAGAAGAGGCT |
| Cct8 (mouse)-R | CCAGGCGATTGATGACCATTT |
| Tcp1 (mouse)-F | CCGCTCCCAGAATGTTATGG |
| Tcp1 (mouse)-R | CGGGATGTTCTACCTCCAGT |
| Msn (mouse)-F | TCTTATGCCGTCCAGTCTAAGT |
| Msn (mouse)-R | GGTCCTTGTTGAGTTTGTGCT |
